# A hybrid metaheuristic algorithm with machine learning for detecting denial-of-service attacks in wireless sensor networks

**DOI:** 10.3389/frai.2026.1738152

**Published:** 2026-02-06

**Authors:** Ashwani Prasad, Karmel Arockiasamy, Kanimozhi Gunasekaran

**Affiliations:** 1 School of Computer Science and Engineering, Vellore Institute of Technology, Chennai, Tamil Nadu, India; 2 Centre for Smart Grid Technologies, Vellore Institute of Technology, Chennai, Tamil Nadu, India

**Keywords:** cybersecurity, denial-of-service attacks, feature selection, genetic algorithm, grasshopper optimization algorithm, hybrid GOA-GA algorithm, intrusion detection system, machine learning

## Abstract

Denial-of-service (DoS) attacks pose a major threat to various kinds of computer networks. There are several kinds of networks that are victims of DoS attacks, one of them being the wireless sensor network (WSN). The main objective of this work is to detect such attacks in wireless sensor networks. These networks are susceptible to intrusion attacks because of their fragile defense mechanisms in unattended environments. Thus, a suitable intrusion detection system must be created to optimally detect DoS attacks and prevent them. This work proposes a hybrid technique called Grasshopper Optimization Algorithm-Genetic Algorithm (GOA-GA), which combines the advantages of two metaheuristic algorithms, namely, the Grasshopper Optimization Algorithm and the Genetic Algorithm, to optimize feature selection based on the given WSN dataset. After optimal feature selection and training, the machine learning classification algorithms classify whether the traffic is normal or benign in the form of four types of DoS attacks, namely, Blackhole, Scheduling, Flooding, and Grayhole attacks. The proposed model and algorithms used are further validated and compared based on standard performance metrics. The experiments conducted during the research show that the GOA-GA method, when combined with the KNN classifier, achieves an accuracy of 95.51% and a recall of 95.51%, exhibiting competitive performance relative to recent state-of-the-art approaches while reducing feature dimensionality and computational overhead. These results indicate that the proposed hybrid optimization strategy offers a robust and efficient solution for DoS attack detection in WSNs, contributing to ongoing research in information security.

## 1 Introduction

Modern society is heavily reliant on information and numerous types of communication technologies for sharing data. With increased usage, accessibility, and popularity of the Internet, several networks, wired or wireless, have become more vulnerable to a wide variety of cyberattacks, especially over the last few decades. The proposed work focuses its study on a special type of such vulnerable wireless network known as the Wireless Sensor Network (WSN). They are comparable to wireless *ad hoc* networks (Di Pietro et al., 2014) in that they depend on a wireless connection and the emergence of networks on their own to enable the wireless transmission of sensor data. Pressure, sound, and other environmental factors are all monitored by Wireless Sensor Networks (WSNs). Modern WSNs are bi-directional and simultaneously collect data (Di Francesco et al., 2011). These networks have become more significant as a study topic because of their multiple real-time applications in crucial military surveillance, battlefields, building security monitoring, monitoring forest fires, healthcare, and other useful environmental applications (Kandris et al., 2020). Achieving the objective of protecting WSNs from different security threats becomes a major challenge because of their constrained resources, including limited battery energy, memory, and processing capabilities (Butun et al., 2014).

**Figure 1:**
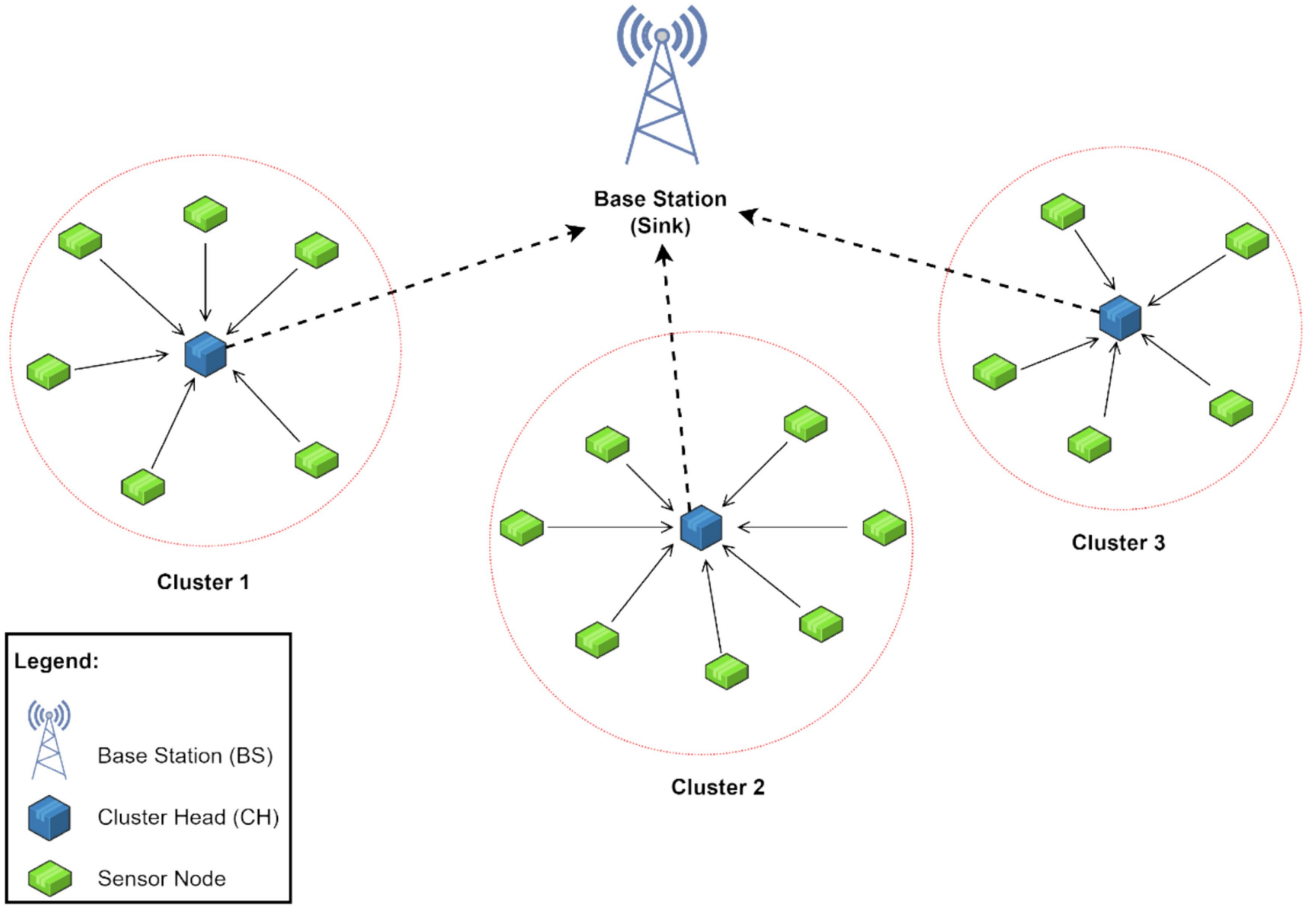
A simple illustration of the node structure of a WSN in the LEACH routing protocol.

The creation of WSNs was made possible by developments in hardware manufacturing, wireless communications, micro-electro-mechanical devices, and information processing. A WSN is composed of several autonomous sensor nodes (SNs) that are scattered throughout different regions of interest to gather crucial data and jointly transfer it wirelessly to a more powerful node known as the sink node or base station (BS). There could be more than one base station in a WSN. The data transmitted across the network depends on specialized WSN protocols. Some of the well-known examples of WSN protocols are TEEN, APTEEN, LEACH, and PEGASIS (Khan et al., 2016). A few recent and energy-efficient protocols, such as the HEESR and DLCP protocols, have also been proposed (Ibrahim Khalaf and Muttashar Abdulsahib, 2020). This study shall focus on the WSNs following the LEACH (Low-Energy Adaptive Clustering Hierarchy) protocol (Heinzelman et al., 2000), particularly the LEACH-C, which is the centralized LEACH protocol. Moreover, there are several variants of the same protocol, such as LEACH-TLCH, V-LEACH, LEACH-H, LEACH-DCHS, etc., which have been studied as well (Fu et al., 2013; Arora et al., 2016; Varshney and Kuma, 2018). Most nodes communicate to cluster heads (CHs) via the hierarchical protocol LEACH, and the cluster heads then compile and pass the data to the base station. In order to predict whether a node will become a cluster head in a particular round, each node runs a stochastic algorithm. This protocol assumes that each node has a radio capable of directly connecting to the base station or the closest cluster head, but that continuous utilization of this radio at full power would be energy inefficient. Figure 1 illustrates the LEACH protocol configuration through a simple WSN node structure having three clusters and a single base station.

WSNs need to be protected against intrusion in order to stop hackers from falsifying sensor data or impeding the delivery of accurate sensor data. The majority of the routing protocols for WSNs optimize for the network’s application-specific nature, the network’s application-specificity, and the limited capabilities of its nodes, but they do not take into account the security aspects of the protocols. It is crucial to examine these protocols’ security characteristics, even though security was not a primary consideration when they were being built (Majumdar and Sarkar, 2015). Besides the security issues with the protocols, there are other issues and challenges associated with WSNs, including hardware and software issues, MAC layer issues, fault tolerance, and robustness (Sharma et al., 2014). Due to the nature of such networks, traditional security measures like encryption might not always be sufficient (Bukhari et al., 2024; Nguyen et al., 2025). Therefore, a strong security measure such as an Intrusion Detection System (IDS) is required. The role of an IDS is to detect and notify users of system or network intrusions. However, because of the constrained resources of the WSN nodes, developing IDSs for WSNs presents a unique difficulty. In order to increase the lifespan of a sensor network, IDS solutions should aim to reduce the battery usage of the sensor nodes. Designing an IDS that can identify an intruder that uses unknown attacks with a high degree of accuracy is difficult. It is also difficult to create the same IDS with a lightweight profile so that the infrastructure of WSNs is not burdened (Doddapaneni et al., 2012; Ghosal and Halder, 2013). Thus, an IDS for a WSN should be carefully designed considering the aforementioned research challenges.

The most prevalent and dangerous cyberattacks that pose a threat to the security of WSNs are those known as denial-of-service (DoS) attacks. The primary goal of these attacks, which come in a variety of forms, is to disrupt or restrict the services offered by WSNs (Farooq et al., 2014; Sen, 2016). The purpose of this work is to develop an IDS that can precisely identify several kinds of commonplace DoS attacks, such as flooding, scheduling, blackhole, and grayhole attacks, to improve the security of WSNs. The motivation behind this study is to address the particular security challenges that WSNs face because of their resource constraints. This research proposes a novel hybrid metaheuristic algorithm called GOA-GA approach that combines the complementary strengths of the Grasshopper Optimization Algorithm (GOA) and the Genetic Algorithm (GA). GOA provides effective global exploration of the search space, while GA enhances exploitation through crossover and mutation operations. By integrating these mechanisms, the proposed GOA–GA algorithm aims to achieve more stable convergence toward informative and compact feature subsets, thereby improving intrusion detection performance without imposing excessive computational burden. As a result, this optimizes feature selection and improve attack classification accuracy, in contrast to standard approaches that frequently ignore the security elements of WSN protocols. Recent reviews of nature-inspired metaheuristic algorithms show a rising trend in hybridization techniques (Rani et al., 2024), supporting the relevance of our GOA–GA hybridization for WSN IDS. This lightweight design is well suited to WSN intrusion detection, where achieving high accuracy with reduced feature dimensionality is essential for real-world applicability.

Hence, based on the above motivation, the SMART (abbreviation for Specific, Measurable, Achievable, Relevant, and Time-Bound) major contributions of this paper are listed as follows:

1. *Specific*: To develop and demonstrate a methodology for detecting Denial-of-Service (DoS) attacks in Wireless Sensor Networks (WSNs) using machine learning and metaheuristic algorithms.
2. *Measurable*: To propose and evaluate a novel hybrid algorithm, GOA-GA (Grasshopper Optimization Algorithm cum Genetic Algorithm), for optimizing feature selection in intrusion detection systems.
3. *Achievable*: To accurately classify and differentiate between multiple types of DoS attacks, including Blackhole, Grayhole, Flooding, and Scheduling attacks, that threaten WSNs.
4. *Relevant*: To validate the proposed methodology with extensive experiments, using standard validation techniques and performance metrics to ensure robustness and reliability.
5. *Time-Bound*: To complete the development, implementation, and validation of the proposed IDS within a predefined timeframe, ensuring timely results and conclusions.

The rest of this research paper is structured as follows: Section 2 provides the background and a thorough literature review on strategies adopted so far to tackle cyberattacks in WSNs and similar networks, with their merits and demerits. This section also summarizes the different datasets and simulation tools used for developing IDS for WSNs against various threats. Section 3 presents the framework for the proposed methodology, discusses the algorithms and software tools used during the research, as well as the limitations and potential challenges, and offers some insights into the model interpretability and practical implications of the proposed solution. Section 4 describes the WSN dataset and DoS attacks, along with the implementation details and the incorporated validation techniques. Section 5 illustrates the experimental results so obtained and discusses the importance of the achieved results while comparing the proposed hybrid algorithm with that of the standard algorithms. Section 6 discusses the limitations and the potential challenges of the proposed methodology. The conclusions of the work are presented in Section 7, along with suggestions for future work.

## 2 Literature survey

In this section, the background pertaining to WSNs and related works associated with WSN security against cyberattacks are discussed. Section 2.1 gives an overview of a typical IDS in a WSN, discusses machine learning, metaheuristic approaches, and deep learning techniques incorporated by several studies to protect WSNs from cyber threats. Section 2.2 does an additional literature review, further highlighting the drawbacks, and provides a comparative summary of the related works.

### 2.1 Background

**Figure 2:**
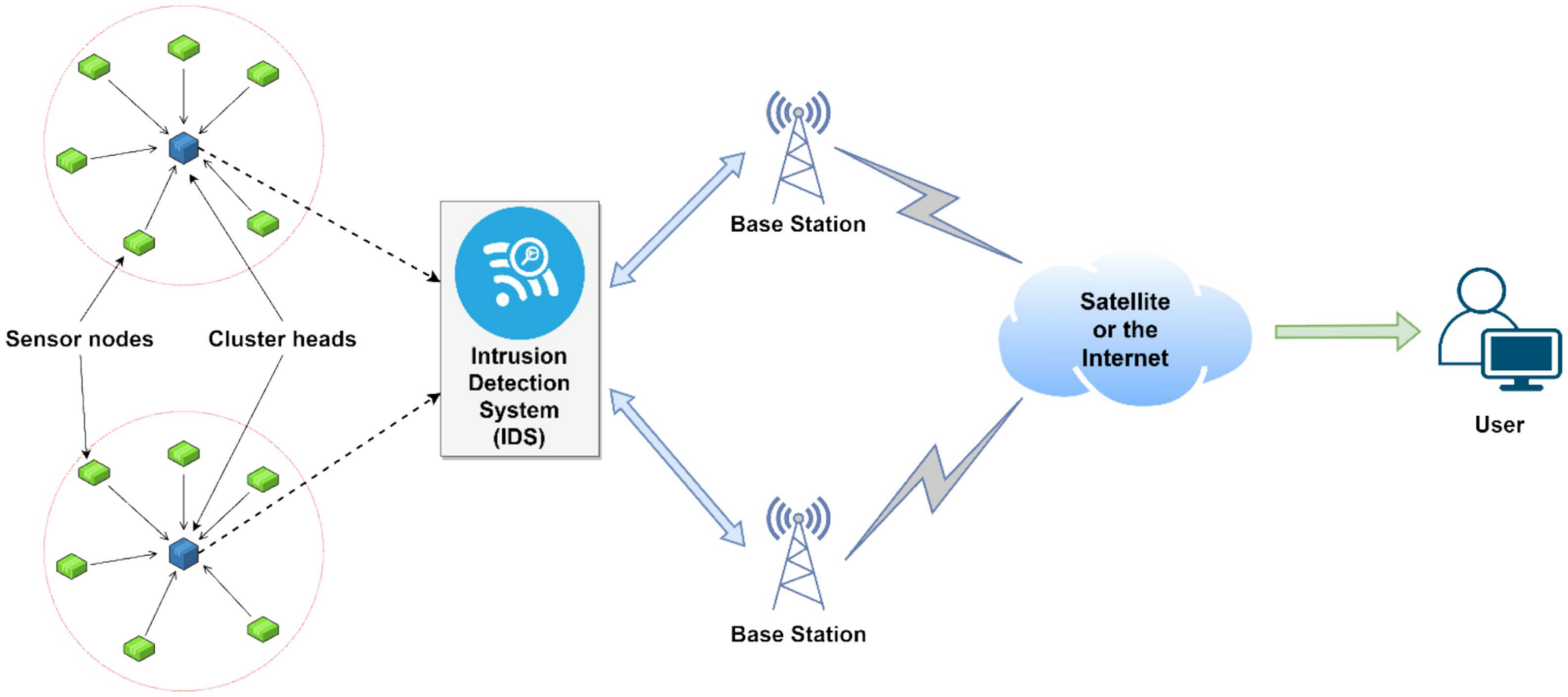
A typical WSN model integrated with an intrusion detection system (IDS), adapted from the general architecture described in the literature.

Wireless sensor networks have become a prominent piece of wireless technology because of their numerous real-life applications. However, because of their susceptible nature, a lot of research has been done and is still going on in building an efficient and lightweight intrusion detection system for WSNs to increase their security. Figure 2 shows a simple archetype model of a WSN with two clusters, two base stations, and an IDS to filter the data sent out by the cluster head to the base station so that reliable data can reach the user end safely. Most of the research studies concerning the development of an IDS for a WSN follow or have proposed a similar architecture as seen in Figure 2.

Machine Learning (ML) has been widely used for the detection of several types of cyberattacks, including DoS attacks in WSNs. Al-Issa et al. (2019) proposed well-known ML algorithms, namely Decision Tree and Support Vector Machine (SVM), to detect attack signatures on a specialized dataset created by them. Though their approach was less costly and less complex, limited attack scenarios and protocols were considered. Next, Almomani et al. (2016) created the WSN-DS dataset and used Multilayer Perceptron artificial neural networks to detect four types of DoS attacks, viz. Grayhole, Blackhole, Flooding, and Scheduling attacks. The results achieved were appreciable but could have been compared with other state-of-the-art techniques as well. In a similar context, the authors in Alsulaiman and Al-Ahmadi (2021) used the same dataset and evaluated the performance of five popular ML algorithms, viz. Naïve Bayes (NB), SVM, Random Forest, J48, and K-Nearest Neighbors (KNN). A comprehensive evaluation was carried out for the algorithms in this paper, but they lacked optimization during feature selection. Zhang et al. (2020) suggested a hierarchical intrusion detection model that groups a WSN’s nodes according to their roles to decrease the energy consumption of nodes during detection processing. The use of the kernel extreme learning machine’s classification algorithm in conjunction with the Mercer Property to synthesize multi-kernel functions is taken into consideration in this study. Furthermore, in Lakshmi Narayanan et al. (2022), the Enhanced Code-based Round-Trip Time (EC-BRTT) method is used to prevent blackhole and wormhole attacks in WSNs with the help of the ML-based Naïve Bayes classifier. The key benefit of the suggested approach was a decrease in communication overhead.

Metaheuristic algorithms, inspired by natural processes, have been widely applied in optimization tasks due to their balance of exploration and exploitation. Foundational works, such as Yang (2020), provide a comprehensive theoretical framework for nature-inspired optimization algorithms, offering insights into their hybridization potential for complex domains like WSN intrusion detection. Building on this theoretical foundation, nature-inspired evolutionary algorithms have come in handy for quite a few researchers in optimizing feature selection while building intrusion detection models for WSNs and similar wireless networks. For instance, Vijayanand et al. (2018) proposed a novel IDS with Genetic Algorithm with tournament-based feature selection and multiple SVM classifiers for wireless mesh networks. Although the suggested model exhibited a high accuracy for attack detection with strong validation against multiple datasets, the working of the proposed method might be indeterminate for WSNs, and further research might be required. Next, the authors in Zhang et al. (2020) studied a special type of WSN known as Mobile Wireless Sensor Networks (MWSNs). The lifetime optimization model for the MWSN is developed in this research using five evolutionary computing (EC) techniques. The benefits and drawbacks of these five techniques for solving the model are examined through numerical simulations. However, the applicability of this model in detecting cyberattacks is unknown.

A rather sophisticated approach has been adopted in Davahli et al. (2020) wherein the model proposed, known as GABGWO, combines the ideas of Genetic Algorithm (GA) and Grey Wolf Optimizer (GWO) mathematical equations to create a support vector machine (SVM)-based lightweight IDS (LIDS). It is also determined that the performance of this hybrid algorithm is superior to that of pure GA, GWO, and other modern approaches. Other hybrid IDS approaches, such as combining Grey Wolf Optimization with SVM, have shown improved detection performance in WSNs (Safaldin et al., 2021). Our GOA–GA approach similarly leverages hybridization but targets faster convergence and better feature minimization.

Deep Learning (DL), which is essentially a subset of machine learning, has been employed by researchers to make security systems for WSNs. For example, in Salmi and Oughdir (2022), the authors used a combined technique called Convolutional Neural Network and Long Short-Term Memory (CNN-LSTM) to detect and classify DoS intrusion attacks on a WSN dataset. The results obtained from this hybrid deep-learning model indicate its high efficiency and also make the model easy to comprehend. Though the model could have been compared with other existing systems as well in the study. Moreover, Ramesh et al. (2021) proposed an optimized Deep Neural Network algorithm for detecting DoS attacks in Wireless Multimedia Sensor Networks (WMSNs). Although their implementation might be intricate, they used consistency-based and correlation-based feature selection along with Multilayer Perceptron (MLP) and Stochastic Gradient Descent (SGD) to achieve highly useful outcomes. Further, to prevent attacks on WSNs, the study done in Pawar and Anuradha (2023) implements an optimized LSTM model for attack detection and prevention based on the fitness rate-based Whale Optimization Algorithm (FR-WOA). This is how this paper sought to optimize multi-objective functions as intended. The investigation revealed that the optimized LSTM’s accuracy is superior to that of traditional LSTM, and the energy consumption of FR-WOA is superior to other evolutionary algorithms.

### 2.2 Related works

Besides machine learning, deep learning, and evolutionary computing techniques, several other logical methodologies have also been adopted by researchers to detect cyberattacks in WSNs for reliable data transmission. An effective trust-based attack detection module is described in Anand and Vasuki (2021) to identify DoS attacks such as selective forwarding and flooding attacks. Although a limited number of DoS attacks were considered for the analysis, the proposed attack detection model performed better than traditional detection methods. Next, Dhamodharan et al. (2022) discussed how Distributed Denial-of-service (DDoS) attacks impair the network’s functionality and the data being transmitted. To manage the attack proactively, the authors presented the Centralized Detect Eliminate and Control (CDEC) algorithm for authorization and a centralized monitoring component. This study emphasized the security and privacy of WSNs, but the network considered during the experiment was small. Similarly, Dhuria and Sachdeva (2018) presented two novel and effective methods to deal with DDoS attacks in WSNs. The first was a lightweight two-way authentication method that would shield WSNs from the majority of attacks, and the second was a traffic analysis-based data filtering method that would identify and shield WSNs from DDoS attacks. Furthermore, Pajila et al. (2022) used a fuzzy logic approach to quickly identify DDoS (Flooding) attacks and retrieve sensor node data. This Fuzzy-Based Detection and Recovery (FDBR) method saved energy and worked better than other similar schemes. But the drawback of this method is that DDoS attacks might not be mitigated in the early stages. Moving on, Altaf Khan et al. (2022) developed a unique method for distinguishing DDoS attacks from the flash crowd (FC) in data traffic by using a Bayesian model to detect aberrant data traffic in WSNs. The proposed novel mechanism is called DDoDF, and the simulation results were obtained by using realistic datasets. The drawbacks of this work include the negligence in considering payload patterns and hop count information. Further on, in Alaparthy and Morgera (2018) an attempt is made to protect a WSN utilizing an immunity theory technique known as Danger Theory. In other words, a multi-level IDS is created based on the characteristics of different immune cells. This technique is well-thought-out and shows a high degree of reliability in detecting DoS and DDoS attacks in WSNs. Last but not least, in Osanaiye et al. (2019) the authors suggested a feature selection approach that combines the three filter methods of Gain ratio, Chi-squared, and ReliefF (triple-filter) for a typical IDS to protect WSNs. As a result, system complexity would decrease and classification accuracy would rise. Additionally, the major advantage of this approach is that the total energy consumed by the sensor nodes during intrusion detection is decreased.

Apart from the significant contributions of the research works in the literature review, they have some limitations as well, which bring into the picture the following research gaps:

1. Very few computer-generated WSN datasets for DoS detection have been developed, and meager research has been conducted on the same, wherein hybrid metaheuristic algorithms with ML classification algorithms have been implemented.
2. Most of the related works used a standalone Genetic Algorithm in the intrusion detection system, except for a few.
3. The evaluation of the proposed model for IDS in WSNs might not have received enough emphasis.

Solving these drawbacks forms the motivation of this research work. Hence, in this work, a new combined approach using the Grasshopper Optimization Algorithm and Genetic Algorithm (GOA-GA) has been proposed for feature selection. In addition, several ML classifiers such as Classification and Regression Tree (CART), KNN, Logistic Regression (LR), and MLP are used and compared to decide which algorithmic combination yields the best DoS attack detection. The hybrid model employed in this research work has also been given careful consideration, and before real-time usage, it would have undergone a thorough evaluation and comparison. Finally, the dataset used in this paper is that of WSN-DS, which was first used in Almomani et al. (2016) and has been researched by various other authors, as seen in Table 1. This shows that the dataset used is authentic for studying DoS attack detection in WSNs.

**Table 1:** Summary of the literature review for various intrusion detection systems used in WSNs.

Sr. No.	References	Year	Dataset	Attacks analyzed	Detection/prevention technique	Accuracy	Recall (detection rate)	Simulation tool	Limitations
1	Al-Issa et al. (2019)	2019	WSN-DS	Blackhole, Flooding, Scheduling, and Grayhole attacks	Decision Tree; Support Vector Machine	Decision Tree: 0.997 SVM: 0.973	Decision Tree: 0.997 SVM: 0.971	WEKA toolbox	Focused only on limited attack types; results dataset-specific, not validated on real deployments
2	Alsulaiman and Al-Ahmadi (2021)	2021	WSN-DS	Blackhole, Flooding, Scheduling, and Grayhole attacks	Naïve Bayes; Support Vector Machine; Random Forest; J48; K-Nearest Neighbors	99.72%	Highest average recall: 0.997	WEKA toolbox	Evaluation limited to a single dataset; scalability and energy efficiency in real WSNs not addressed
3	Zhang et al. (2020)	2020	NSL-KDD; UNSW-NB 15	DoS, Probe, R2L, and U2R attacks	Multi-Kernel Extreme Learning Machine (MK-ELM)	98.3% overall	98.03% for DoS attacks	MATLAB R2014b version	Used generic datasets (not WSN-specific); applicability to constrained WSN environments uncertain
4	Lakshmi Narayanan et al. (2022)	2021	Not specified	Blackhole attacks; Wormhole attacks	Naïve Bayes; Enhanced Code-based Round Trip Time (EC-BRTT)	EC-RTT: 0.91 for 100 nodes	EC-RTT: 0.91 for 100 nodes	NS-2	Dataset not specified; tested on small-scale scenarios; lacks validation on diverse topologies
5	Davahli et al. (2020)	2020	KDDcup99	Not specified	Genetic Algorithm; Grey Wolf Optimizer	99.09%	99.30%	WEKA toolbox	Relies on the outdated KDDcup99 dataset; lacks validation against modern WSN-specific attacks
6	Salmi and Oughdir (2022)	2022	WSN-DS	Blackhole, Flooding, Scheduling, and Grayhole attacks	Convolutional Neural Network; Long Short-Term Memory	0.944	0.922	Python 3.7.7, Python (Google Colab)	High computation and energy cost; not suitable for resource-constrained WSN nodes
7	Pawar and Anuradha (2023)	2023	Created and used an experimental dataset	Blackhole attacks; Wormhole attacks	Long Short-Term Memory; Fitness Rate-based Whale Optimization Algorithm (FR-WOA)	Not specified	Not specified	Python	Results dataset-specific; no standard dataset used; performance comparison with benchmarks missing
8	Anand and Vasuki (2021)	2021	Not specified	Selective Forwarding attacks; Flooding attacks	Multi-dimensional Trust Parameters	Not specified	Between 95 to 100%	NS-2.33	Limited attack types analyzed; energy overhead of trust calculation not considered
9	Dhamodharan et al. (2022)	2021	Not specified	DDoS attacks	Centralized Detect Eliminate and Control (CDEC) Algorithm	Not specified	Not specified	NS-2.34	Centralized approach—single point of failure; scalability in large WSNs questionable
10	Dhuria and Sachdeva (2018)	2018	Not specified	DDoS attacks	Two-Way Authentication Method; Traffic Analysis-Based Data Filtering Method	Not specified	Not specified	NS-2	Lacks experimental dataset validation; energy consumption overhead is not studied
11	Pajila et al. (2022)	2022	Not specified	DDoS attacks	Fuzzy-based DDoS Attack Detection and Recovery Mechanism (FBDR)	Not specified	Close to 99% as per the given graph	MATLAB	Performance validated only via simulation graphs; lacks comparison with ML/DL models
12	Alaparthy and Morgera (2018)	2018	Not specified	Blackhole, Wormhole, DDoS and Selective Forwarding attacks	Danger Theory; Artificial Immune System	Not specified	Not specified	Cooja	High algorithmic complexity; computational cost unsuitable for low-power WSN nodes
13	Osanaiye et al. (2019)	2019	NSL-KDD	DoS, Probe, R2L, and U2R attacks	Three filter methods, namely, Gain Ratio, Chi-Squared, and ReliefF (Triple-Filter)	99.67%	99.76%	WEKA toolbox	Based on the generic NSL-KDD dataset, not validated on real WSN traffic; ignores energy/resource constraints

## 3 Proposed methodology

In this section, the methodology behind this research work is explained in detail. Section 3.1 presents the solution architecture of DoS attack detection in WSNs from the dataset provided. Section 3.2 explains the metaheuristic algorithms used in the study, including the hybrid algorithm. Section 3.3 explains the machine learning classifiers used for training and classification purposes. Lastly, Section 3.4 provides insights into the model’s interpretability and practical implications.

### 3.1 Architecture

**Figure 3:**
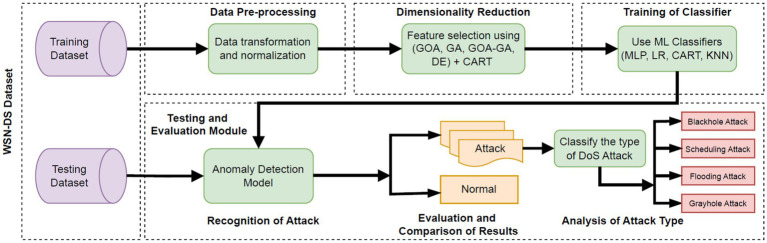
Proposed solution architecture to detect DoS attacks in a WSN based on a given dataset.

This paper presents a solution wherein an IDS can detect cyber threats like DoS attacks in WSNs by following a proposed architecture, as observed in Figure 3. Figure 3 illustrates the process of dividing the dataset into training and testing sets, then pre-processing the data, followed by dimensionality reduction and training of classifiers, and ultimately building the anomaly detection model to classify the results as either normal or indicate the presence of an attack. In case of an attack, the attack can be further classified into four types as per the information present in the dataset. The following architecture is inspired by the work done in Dwivedi et al. (2020).

The implementation of this research work is based on four modules in total, which are in accordance with Figure 3. Firstly, in the data pre-processing module, four sub-steps are involved:

- *Loading and Inspection*: The dataset is imported as a data frame, and its structure is studied.
- *Feature Analysis*: Attributes are examined and separated into input features and target labels.
- *Data Transformation*: Necessary normalization or scaling is applied to ensure uniform feature distribution.
- *Balancing*: If the dataset is imbalanced, resampling techniques (e.g., oversampling, undersampling) are applied to achieve class balance.

Next, in the second module, the dimensionality reduction is achieved through optimized feature selection using metaheuristic algorithms such as the Grasshopper Optimization Algorithm, Genetic Algorithm, Differential Evolution, and the proposed hybrid approach. Dimensionality reduction is important as it simplifies models by decreasing input features, which improves efficiency and performance, mitigates overfitting, enhances accuracy, and interpretability. This approach makes it easier to handle high-dimensional data, speeds up computation, helps models generalize better, and makes them easier to understand, particularly in real-time applications like detecting DoS attacks in WSNs. An optimized decision tree (CART) algorithm is used as the fitness function in all four metaheuristic algorithms required for feature selection. CART was selected because it offers interpretable feature importance, efficient computation on high-dimensional data, and robustness to mixed-type attributes and class imbalance. Its splitting criteria, based directly on classification accuracy (e.g., Gini impurity), make it a practical and meaningful metric for guiding optimization. Recent studies reinforce its effectiveness: Velasco-Mata et al. (2021) demonstrated high F1 performance with small feature subsets evaluated via decision trees, and other works validate its role as a strong baseline compared to more complex methods like Boruta-Random Forest in WSN IDS scenarios (Subbiah et al., 2022). The broader literature emphasizes the value of CART’s transparency and performance in security applications (Li et al., 2024; Emirmahmutoğlu and Atay, 2025). The output of this second module would be the best subset of input features based on which the model shall be trained for optimal results. Only the top 4 features from the dataset are decided to be used for further use by ML classifiers to reduce the overhead on the WSNs. A point to note here is that feature selection using these four metaheuristic algorithms was performed exclusively on the training data to avoid introducing information from the test set during the optimization process.

In the third module, the training of ML classifiers takes place. ML classification algorithms such as LR, CART, KNN, and MLP are used in the training of the models, with each of the metaheuristic algorithms used. So, in total, there are 16 combinations of metaheuristic algorithms and ML classifiers. To validate the training performance, cross-validation techniques could be used. Next, in the fourth module, the trained models are tested against the test dataset. The models are further evaluated based on well-known performance measures and finally compared with each other based on these metrics. Thus, from the results, we could conclude which model(s) are proficient in predicting DoS attacks in WSNs.

### 3.2 Hybrid GOA-GA and other metaheuristic algorithms

The main proposed methodology in this research work lies in the hybrid GOA-GA approach. Apart from that, the other three metaheuristic algorithms were also used during the experiment and hence are discussed below.

#### 3.2.1 Grasshopper optimization algorithm

The Grasshopper Optimization Algorithm (GOA) is a cutting-edge, effective metaheuristic algorithm inspired by grasshoppers. There are two stages in the grasshopper life cycle: nymph and adult. While the swarm expands slowly during the nymph phase, it expands quickly during the adult phase, taking large steps (Fathy, 2018). GOA, which was modeled after a metaheuristic based on nature, can be used in two stages: exploration and exploitation. Swarms move quickly during the exploration phase, but only locally during the exploitation phase. Numerous researchers have become interested in it to find solutions to numerous real-world problems as a result of the vast investigation and quick convergence. Therefore, GOA has been used in this work because of such advantages and also for assessing its applicability in feature optimization for WSNs. The mathematical model of GOA (Ewees et al., 2018) which mimics the behavior of grasshoppers consists of the variable $X_{i}$ which denotes the position of the *i*th grasshopper or solution and is given by:


$$X_{i}=S_{i}+G_{i}+A_{i}$$ (1)

where $G_{i}$ is the gravitational pull on the solution, $A_{i}$ denotes wind advection, and $S_{i}\;$ denotes social interaction between the solution and the other grasshoppers. The location of each solution after random behavior has been included is represented by the Equation 2 below:


$$X_{i}=r_{1}S_{i}+r_{2}G_{i}+r_{3}A_{i}$$ (2)

where [0, 1] is the range for the random integers $r_{1}$ , $r_{2}$ , and $r_{3}$ . The social interaction between the solution and the other grasshoppers is represented by the Equations 3, 4 below:


$$S_{i}=\sum_{j=1}^{N}\;s(d_{\mathrm{ij}}){\hat{d}}_{\mathrm{ij}},\text{where}\;i\neq j$$ (3)

$$s=fe^{\frac{-r}{l}}-e^{-r}$$ (4)

where $\hat{d_{\mathrm{ij}}}=\frac{|x_{j}-x_{i}|}{d_{\mathrm{ij}}}\;$ represents the unit vector and $d_{\mathrm{ij}}=|x_{j}-x_{i}|$ indicates the distance between the *i*th and *j*th grasshoppers. Additionally, l is the appealing length scale and f is the degree of attraction, and s reflects the strength of two social factors (repulsion and attraction between grasshoppers). The following Equation 5 demonstrates how to determine the gravitational force $G_{i}$ :


$$G_{i}=-g\hat{e_{g}}$$ (5)

where $\hat{e_{g}}$ is a unit vector pointing toward the earth’s center and g stands for the gravitational constant. How to determine $A_{i}$ is shown in the equation below:


$$A_{i}=u\hat{e_{w}}$$ (6)

where $\hat{e_{w}}$ is the unit vector in the wind direction and u stands for the drift constant. Equations 3–6 are combined to get Equation 1 as follows:


$$\begin{array}{c} X_{i}=\sum_{j=1}^{N}s(d_{\mathrm{ij}})\hat{d_{\mathrm{ij}}}-g\hat{e_{g}}+u\hat{e_{w}}\\ =\sum_{j=1}^{N}s(|x_{j}-x_{i}|)\frac{|x_{j}-x_{i}|}{d_{\mathrm{ij}}}-g\hat{e_{g}}+u\hat{e_{w}},\text{where}\;i\neq j \end{array}$$ (7)

To address optimization concerns, prevent grasshoppers from fast reaching their comfort zone and the swarm from failing to converge to the target site, and solve optimization problems, Equation 7 is adjusted as follows:


$$X_{i}^{d}=c(\sum_{j=1}^{N}c\frac{UB_{d}-LB_{d}}{2}s(|x_{j}^{d}-x_{i}^{d}|)\frac{|x_{j}-x_{i}|}{d_{\mathrm{ij}}})+G+A,\;\text{where}\;i\neq j$$ (8)

where G=0 and A is the best solution in the *d*th dimension, $UB_{d}$ and $LB_{d}$ are the corresponding upper and lower limits in the *d*th dimension. The model for c is mentioned in Equation 9:


$$c=c_{\max}-\text{iter}\frac{c_{\max}-c_{\min}}{\max_{\text{iter}}}$$ (9)

where iter is the current iteration, $\max_{\text{iter}}$ denotes the maximum number of iterations, and $c_{\max}$ and $c_{\min}$ denote the maximum and minimum values of c , respectively. The steps of the Algorithm 1 for GOA are given as follows:

**Algorithm 1:**
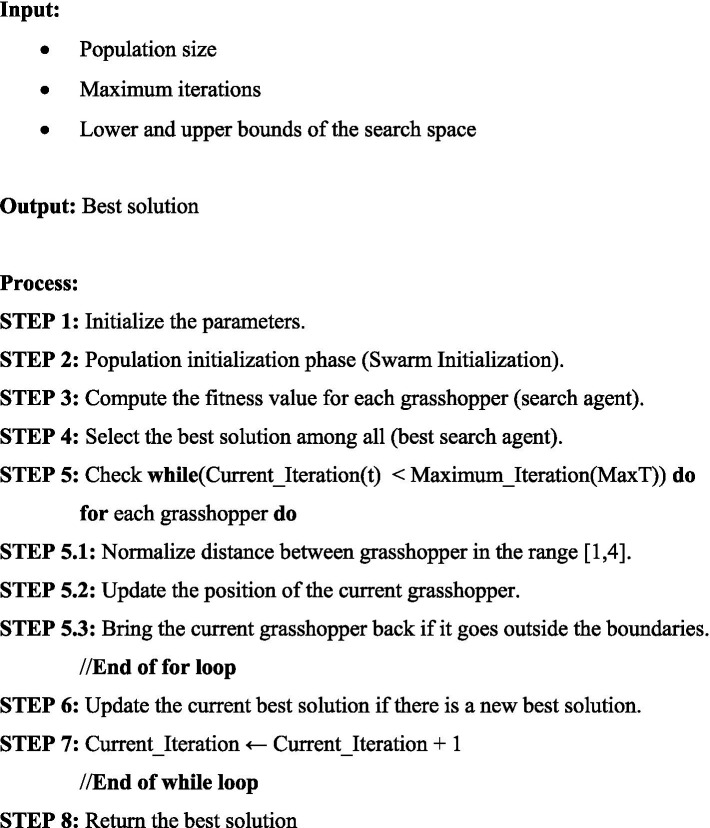
Grasshopper optimization algorithm.

#### 3.2.2 Genetic algorithm

In the larger category of evolutionary algorithms (EA), a genetic algorithm (GA) is a metaheuristic that draws inspiration from the process of natural selection. Utilizing biologically inspired operators such as mutation, crossover, and selection, genetic algorithms are frequently employed to produce high-quality solutions to optimization and search problems (Bhola et al., 2020). Given their ability to handle a high number of characteristics and their effectiveness in swiftly searching through the feature space to find the most pertinent features, genetic algorithms can be employed to identify the most relevant features required for DoS attack detection in WSNs. Additionally, GA is used in this work because they are simple to use and can be integrated with other strategies, like machine learning, to enhance performance. The mathematical model concerning the different phases of GA is presented as follows:

- *Initialization*: Generate an initial population of candidate solutions as mentioned in Equation 10:

$$P_{0}=x_{1}^{0},x_{2}^{0},\ldots ,x_{n}^{0}$$ (10)

where $P_{0}$ is the initial population of candidate solutions; n is the number of individuals in the population; $x_{i}^{0}$ is the i^th^ candidate solution in the initial population.

- *Evaluation*: Evaluate the fitness of each candidate solution:

$$f(x_{i}^{t})\;\text{for}\;\;i=1,2,\ldots ,n$$ (11)

where $x_{i}^{t}\;$ is the fitness of the *i*th candidate solution at generation t .

- *Selection*: Select the best-performing candidates to generate a mating pool:

$$P_{s}\subseteq P_{t}$$ (12)

where $P_{s}\;$ is the mating pool, a subset of the population, and $P_{t}\;$ is the population at generation t .

- *Crossover*: Generate offspring by combining the traits of parents in the mating pool:

$$x_{i}^{t+1}=C(x_{j_{1}}^{t},x_{j_{2}}^{t})$$ (13)

where $x_{i}^{t+1}\;$ is an offspring produced by combining the traits of two parents; C is a crossover operator that takes two parent solutions and generates an offspring solution that combines their traits; $j_{1}\;$ and $j_{2}$ . These are indices of the two parent solutions that are used to generate the offspring solution.

- *Mutation*: Introduce small random changes in some of the offspring:

$$x_{i}^{t+1}=M(x_{i}^{t+1})$$ (14)

where M is a mutation operator that takes an offspring solution and makes a small random change to it, introducing new genetic material that was not present in the parents. The steps of the Algorithm 2 for GA are given as follows:

**Algorithm 2:**
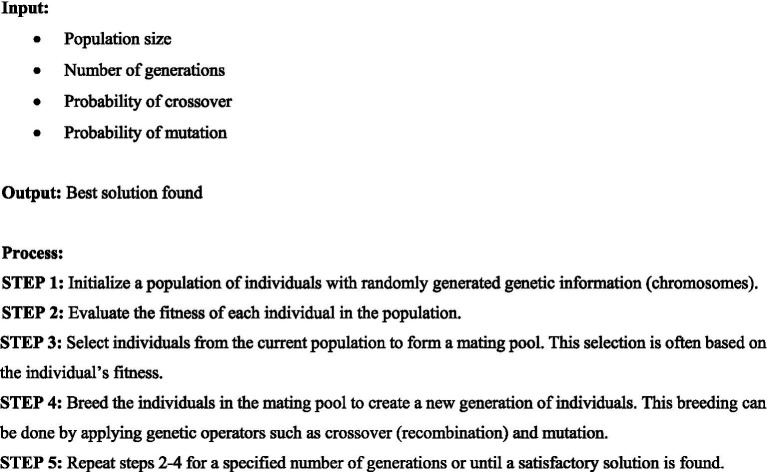
Genetic algorithm.

#### 3.2.3 Hybrid grasshopper optimization algorithm cum genetic algorithm

The GOA-GA is the main and proposed algorithm of this research work. The major reason why this hybridization concept came into the picture with GOA and GA is because of the unique and novel combination that they have. This research work might be one of the first works to propose a hybrid GOA-GA algorithm for feature selection for detecting DoS attacks in WSNs. This hybrid algorithm may be able to overcome the drawbacks of each individual approach and produce better results more quickly by combining the advantages of the two algorithms. Furthermore, using our hybrid approach for feature selection with machine learning models may enhance the precision and effectiveness of identifying DoS attacks in WSNs.

*Rationale for choosing GOA-GA for this study*: The Grasshopper Optimization Algorithm (GOA) is effective for global exploration due to its adaptive social interaction mechanism, but it often suffers from slow convergence and premature stagnation near local optima. Conversely, the Genetic Algorithm (GA) excels at local exploitation through evolutionary operators such as crossover and mutation, yet it may lack strong global search capability and can converge slowly when the search space is large. By hybridizing GOA and GA, we exploit GOA’s strong exploration ability while leveraging GA’s exploitation mechanisms to refine candidate solutions. Thus, GOA and GA compensate for each other’s shortcomings—GOA prevents GA from being trapped in local optima, while GA accelerates convergence by refining GOA’s diverse candidate solutions. This synergy enhances both convergence speed and accuracy, making the hybrid algorithm particularly suitable for high-dimensional feature selection problems in WSN intrusion detection, where balancing exploration and exploitation is critical.

**Figure 4:**
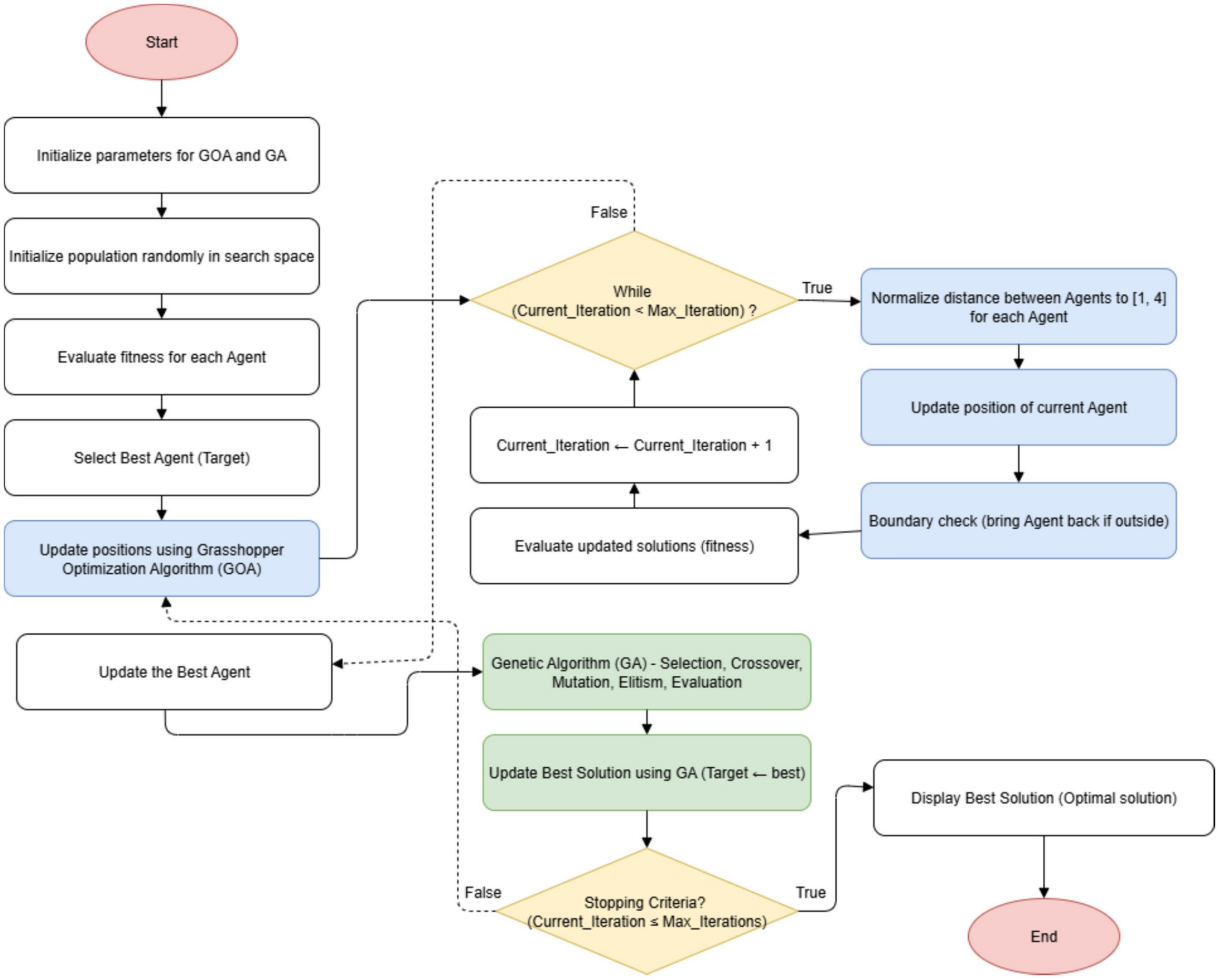
Flowchart of the proposed GOA-GA approach.

The steps of the Algorithm 3 for GOA-GA are given as follows, and the same is depicted in the form of a flowchart in Figure 4.

**Algorithm 3:**
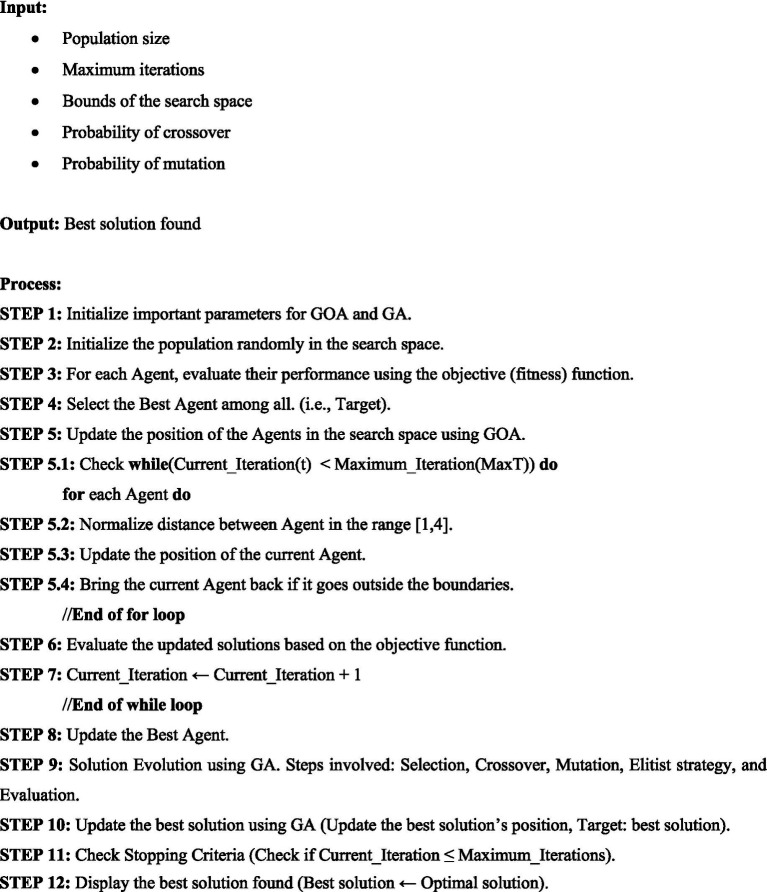
Hybrid grasshopper optimization algorithm cum genetic algorithm (proposed).

In step 5 of the algorithm, the same Equation 8 is used, which was earlier used in GOA for updating the positions of the agents in the search space. Equations 11–14 pertaining to evaluation, selection, crossover, and mutation are employed for Step 9, which involves solution evolution utilizing GA. Thus, the hybridization process mathematically alternates between GOA exploration (Equation 8) and GA exploitation (Equations 11–15) until convergence criteria are met. In addition, the elitist strategy is also included in this step. The elitist selection approach, which is straightforward, makes sure that the best answer so far is always included in the following group of candidate solutions. This helps stop the gradual loss of effective solutions. The elitist strategy can be expressed mathematically as follows:


$$P_{t+1}=P^{\prime }\cup x_{t}^{*}$$ (15)

where $x_{t}^{*}$ is the best solution so far discovered in the optimization process, $P_{t+1}$ is the next generation of candidate solutions, and $P^{\prime }$ is the collection of candidate solutions produced through selection, crossover, and mutation processes. The set union is represented by the operator ∪ .

*Limitations addressed by the proposed hybrid approach*:

- *GOA limitation*: Prone to premature convergence, weak exploitation near optima.
- *GA limitation*: Slow convergence in large search spaces, risk of losing diversity.
- *Proposed hybrid*: GOA ensures a diverse search of the feature space; GA enhances local search and solution refinement; elitist strategy prevents the loss of good solutions. Together, they achieve better feature subset optimization for IDS in WSNs.

#### 3.2.4 Differential evolution

Differential Evolution (DE) is an evolutionary algorithm that was developed by Storn and Price and is an effective, straightforward, and quick global search evolutionary algorithm. The differential mutation operator used by DE, which possesses the properties of search direction and search step-size adaptivity, is what sets it apart from other algorithms the most. DE has the benefits of a straightforward structure, user-friendliness, and high robustness (Zhang et al., 2020; Deng et al., 2021). DE is very similar to GA as both algorithms involve a selection process that identifies the best-performing individuals from the population, and then uses them to generate new candidate solutions for the next iteration. The implementation of DE also has similarities with that of the GA. Due to this reason and for comparison purposes with other metaheuristic algorithms, DE is included in this work. The steps of the Algorithm 4 for DE are given as follows:

**Algorithm 4:**
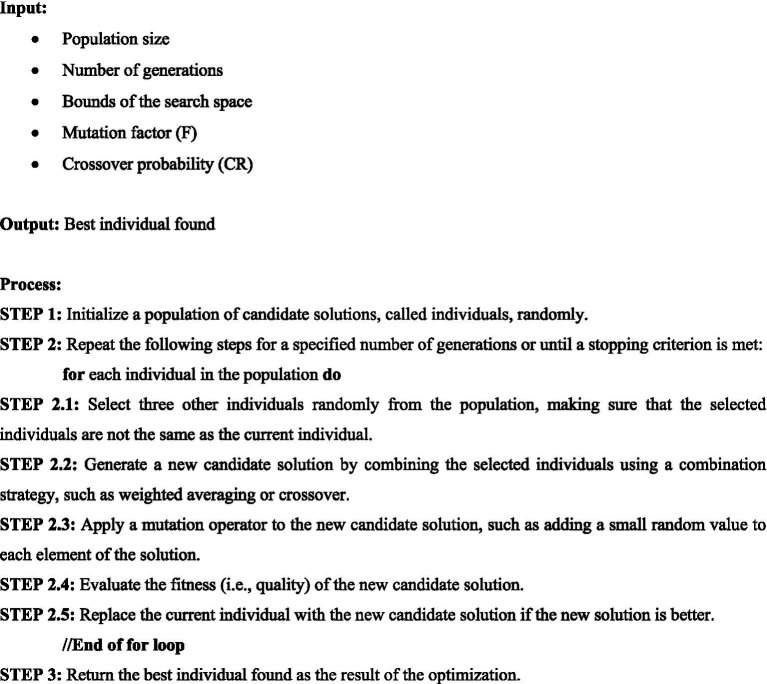
Differential evolution.

### 3.3 Machine learning classifiers

During the experiment study, four of the well-known machine learning classification algorithms were used, which are discussed as follows. Note that each of the following algorithms is a supervised learning algorithm, but at the same time, it is based on different concepts.

#### 3.3.1 Multilayer perceptron

MLP is a type of artificial neural network that consists of at least three layers of neurons: an input layer, one or more hidden layers, and an output layer (Singh and De, 2017). This method’s fundamental strategy is to transform a large number of real-valued inputs into outputs by varying the weights of its internal nodes. During the training of a dataset using the back-propagation learning technique (Singh and De, 2017), MLP obtains a function $f(x):R^{i}\to R^{t}$ , where i , t ∈ Q + represents input and output dimensions separately. The equation for this is given as (Equation 16):


$$y=\delta \{\sum_{i=1}^{m}(w_{i}X+b)\}=\delta (W^{T}X+b)$$ (16)

where δ represents the activation function, w stands for the weight vectors, X for the input vectors, and b for the bias. This neural classifier has been used widely in practice in several disciplines, including pattern classification, identification, and prediction. For this work, the MLP classifier is chosen because MLPs are capable of learning complex, non-linear relationships between the input features and the target variable. This is important in the given research case because it is likely that the input features may not have a simple, linear relationship with the target variable (i.e., the presence or absence of a DoS attack). Also, they are well-suited for detecting patterns and relationships in high-dimensional data, which is often the case in WSNs where there may be many different sensors generating data.

#### 3.3.2 K-nearest neighbors

In KNN classification, all computation is postponed until after the function has been evaluated, and the function is only locally approximated. Since this approach depends on distance for classification, normalizing the training data can significantly increase accuracy if the features reflect several physical units or have wildly different sizes. However, several factors, such as the choice of the *k* value, the choice of distance metrics, and others, can impact how well the KNN classification performs. The distance between two data points x and y in a feature space of d dimensions is given by the Euclidean distance as given in Equation 17:


$$d(x,y)=\sqrt{\sum_{i=1}^{d+1}{\left(x_{i}-y_{i}\right)}^{2}}$$ (17)

which is the most used distance metric in KNN. Indicated as $N_{k}(x,D)$ , the collection of k-nearest neighbors is defined as in Equation 17a:


Nk(x,D)={xiinD∣d(x,xi)≤d(x,xj)forallxjinD,j≠i},where∣Nk(x,D)∣=k

In other words, $N_{k}(x,D)$ is the set of 𝑘 data points in 𝐷 that have the smallest distance to 𝑥. The reason why KNN is used for this research work is that it is simple to implement and can be effective in cases where the decision boundary between classes is not well defined, as seen in the dataset used. This could be the case for detecting DoS attacks, where the patterns of attack may not be easily characterized by a specific model or algorithm.

#### 3.3.3 Logistic regression

Logistic regression is mainly used for classification purposes (Zou et al., 2019). The probability of an event occurring depending on one or more input features can be modeled using this well-liked and often-used classification approach. In the given situation, where machine learning is applied to the WSN dataset to detect DoS attacks, LR can be helpful since it enables forecasting the likelihood that a specific network packet or communication will be used in a DoS attack. In addition to being reasonably simple to understand, LR can shed light on the connection between the input features and the expected chance of a DoS attack taking place. This can help in determining which characteristics are most crucial for spotting DoS attacks in WSNs and can help in the creation of stronger defenses against them. The logistic function has the following formula as given in Equation 18:


$$p(x)=\frac{1}{1+e^{-\frac{\left(x-\mu \right)}{s}}}$$ (18)

where s is a scale parameter and μ is a location parameter (the midpoint of the curve, where p( μ ) = 1/2). Maximizing the likelihood function represents the likelihood that the provided data set was generated by a specific logistic function as given in Equation 19:


$$L=\prod_{k:y_{k}=1}p_{k}\prod_{k:y_{k}=0}(1-p_{k})$$ (19)

when the likelihood function L is used. In the k^th^ observation, $y_{k}$ is the binary answer variable and $p_{k}$ is the anticipated probability of the positive class, or the likelihood that $y_{k}=1$ .

#### 3.3.4 Classification and regression tree

CART is a type of decision tree algorithm used for both classification and regression purposes (Breiman et al., 2017). To construct a decision tree based on the Gini impurity index, the CART method comes into the picture. It offers a wide range of useful applications and is a fundamental machine learning algorithm. As the CART classifier is already used for fitness functions while implementing the metaheuristic algorithms, it is one of the reasons why optimized CART is employed for classification in this research case. It can be advantageous to use the same classifier for all of the classification tasks because it keeps the methodology consistent. As a classification technique, decision trees also have several benefits, including their interpretability and capacity for both categorical and numerical data. Additionally, they can handle huge datasets and train quickly. Decision trees function better when their CART is optimized since it reduces overfitting and increases generalization. The following is a definition of Gini impurity:


$$I_{G}(p)=\sum_{i=1}^{c}p_{i}(1-p_{i})\;$$ (20)

where c is the number of classes and $p_{i}$ denotes the percentage of samples that belong to class i . In CART, the quality of a split is evaluated using the information gain. The decrease in entropy (or Gini impurity) brought on by the split is referred to as information gain. The formula for information gain is as follows:


$$\mathrm{\Delta I}=I(p)-\sum_{j\in \{L,R\}}\frac{N_{j}}{N}I(p_{j})\;$$ (21)

where I(p) is the parent node’s impurity, $N_{j}$ denotes the number of samples in the j^th^ child node, N denotes the total number of samples, and $p_{j}$ denotes the percentage of samples in the j-th child node.

### 3.4 Model interpretability and practical implications

In this section, the interpretability, and practical implications of the IDS in real-world situations are covered. It is discussed here that adaptive learning and frequent upgrades of the IDS could improve its efficacy in addressing evolving cyber threats. Its usefulness can be increased by working with cybersecurity specialists and industry stakeholders to validate it in various contexts. Finally, upholding ethical and data privacy laws will increase the validity and reliability of the results.

#### 3.4.1 Interpretability of the model

The interpretability of machine learning classifiers and hybrid metaheuristic algorithms is still a challenge, despite their ability to detect DoS attacks with high accuracy. Gaining trust and assuring the model’s dependability require an understanding of how it makes decisions. To improve the model’s interpretability, strategies like feature importance analysis, SHAP (SHapley Additive exPlanations), and LIME (Local Interpretable Model-agnostic Explanations) might be used. Network administrators can gain a better understanding of and confidence in the IDS’s decision by receiving insights into the features that have the greatest impact on threat detection.

#### 3.4.2 Practical implications of deploying the IDS

Incorporating the suggested IDS into actual WSNs necessitates considering many pragmatic factors. These consist of the necessary processing resources, the simplicity of integration with the current network architecture, and the possible influence on the overall performance of the network. The suggested approach should be made to use the least number of resources possible while integrating easily with the hardware and communication protocols already in place in the WSN. Furthermore, it’s crucial to make sure the IDS does not add a lot of cost or delay that can interfere with regular network operations.

#### 3.4.3 Adaptive learning and continuous updating

It is essential to include techniques for adaptive learning and constant updating of the IDS due to the dynamic nature of cyber threats. Attack methods and patterns change often; therefore, an inactive IDS can easily become outdated. By putting adaptive learning strategies into practice, such as reinforcement learning and online learning algorithms, IDS may update its detection models in real-time and learn from new attack patterns. This flexibility will improve the IDS’s long-term effectiveness and resistance to new threats. Moreover, applied IDS deployments have already demonstrated tangible benefits in WSN environments. For instance, an IDS integrated with a CLGO-enhanced SVM achieved practical improvements in packet delivery rate and energy consumption (Gupta et al., 2023), underscoring the potential of hybrid optimization and adaptive learning in real-world scenarios.

#### 3.4.4 Collaboration with industry stakeholders and cybersecurity experts

It is essential to collaborate with cybersecurity experts and industry stakeholders in order to validate and improve the suggested methodology. Through industry relationships, access to operational insights, real-world data, and practical difficulties that are not usually met in academic research can be made possible. Working collaboratively to validate the proposed IDS in various operating situations will help find any flaws and make it easier to refine it to comply with industry standards and specifications.

#### 3.4.5 Ethical considerations and data privacy

Developing and implementing intrusion detection systems requires careful attention to ethical issues and data protection laws. The suggested IDS needs to make sure it does not invade user privacy or gather pointless data. Respecting data privacy laws, such as the General Data Privacy Regulation (GDPR), is crucial to keeping the validity and reliability of study findings. The responsible use and deployment of the IDS should also be guided by ethical concerns to make sure that it does not get misused and that neither people nor systems are harmed.

## 4 Experimental analysis and validation

In this section, experimental analysis and validation techniques related to this work’s implementation are discussed. Section 4.1 briefly discusses the experimental setup and tools used. Section 4.2 illustrates the dataset used in the experimental study and its related features. Next, in Section 4.3, the selection of hyperparameters for each of the ML classifiers during their training is discussed. Lastly, Section 4.4 describes the standard performance metrics and validation techniques used during the experiment to evaluate the models.

### 4.1 Experimental setup and tools used

The HP laptop used for this project’s studies has an Intel(R) Core (TM) i5-8265UC processor running at 2.4 GHz, 16.0 gigabytes of RAM, and 256 gigabytes of solid-state drive (SSD) memory storage capacity. The crucial Python modules and Jupyter Notebook 6.5.2, a web-based, interactive computing notebook environment, were installed locally using Anaconda Navigator 2.3.2. The project’s execution took place in the same computing environment, and the notebook’s operating system was Windows 10 (Version 22H2).

Python programming (version 3.9.16) was used for the entire project’s implementation and coding. Throughout the implementation, crucial libraries including pandas, NumPy, Matplotlib, scikit-learn, and DEAP were used.

### 4.2 The WSN-DS dataset

**Figure 5:**
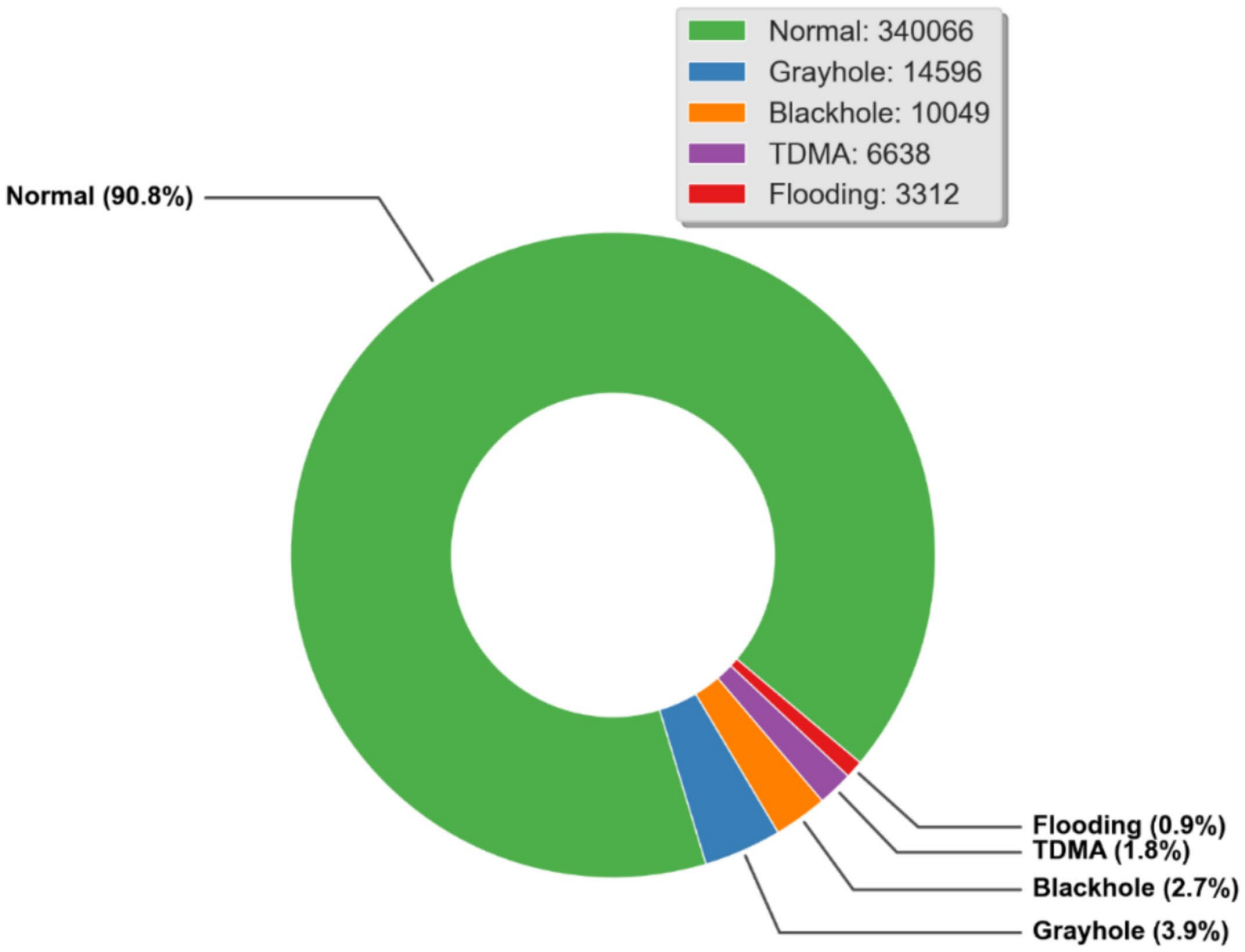
The WSN-DS dataset’s “Attack type” attribute’s composition.

The dataset utilized in this research work is known as WSN-DS and was cited in Almomani et al. (2016). Essentially, it is a dataset for wireless sensor networks used by intrusion detection systems. 374,661 records and 19 columns make up the dataset. It was discovered during data pre-processing that the dataset did not contain any null or NA values. Figure 5 shows the distribution of the data points together with the count value for each class label.

The attributes of the dataset are split into input/independent variables and a target/output variable since the categorization of class labels is the ultimate objective. The following variables (18 in number) were included in the input: “id,” “Time,” “Is_CH,” “who CH,” “Dist_To_CH,” “ADV_S,” “ADV_R,” “JOIN_S,” “JOIN_R,” “SCH_S,” “SCH_R,” “Rank,” “send_code,” “DATA_S,” “DATA_R,” “Data_Sent_To_BS,” “dist_CH_To_BS” and “Expaned Energy.” The “Attack type” attribute made up the target variable.

Ultimately, 4 out of the 18 input features were chosen in order to lessen the burden on WSNs and lower the number of features required to forecast attacks. A great compromise between high model performance and computational efficiency has been demonstrated by the thorough experimentation and cross-validation that supported this decision. A model’s capacity to be applied to new data must be maintained by preventing overfitting, which is achieved by choosing four features. The interpretability of the model is also improved, and the influence of each feature on DoS attack detection is easier to comprehend with a reduced feature set. With respect to the restricted processing power and energy constraints typical of WSNs, this method greatly minimizes computational complexity and resource usage. A small feature set of this size provides a good trade-off between performance and complexity. The hybrid GOA-GA algorithm effectively identified these four features namely, “send_code,” “Time,” “Data_Sent_To_BS,” and “JOIN_R” as the most relevant and impactful subset, ensuring a robust and efficient detection mechanism without redundancy.

While the proposed GOA-GA approach demonstrated strong classification performance, metaheuristic algorithms are inherently stochastic and can yield different feature subsets across runs. To evaluate the stability of our selected features, we performed multiple independent runs (*N* = 20) and computed the Jaccard similarity coefficient between the resulting subsets. Across repeated runs, the average pairwise Jaccard similarity of selected feature subsets was approximately 0.70, indicating that while minor variations occurred, the algorithm consistently converged on a stable core set of features. This suggests that the algorithm consistently prioritized a core group of features, with minor variation in less informative attributes. This aligns with observations in prior studies on stochastic feature selection (Yang, 2020).

The dataset is divided 8:2 in ratio. Accordingly, 80% of the dataset will be used for training and the remaining 20% for testing. The rationale for this ratio’s selection is to ensure that the models receive a sufficient number of samples during the training phase, allowing them to make as accurate a classification prediction as feasible for the testing set. Additionally, the usual ratio used in the majority of the research reviews is 8:2. The overall data count for the various classes for the training and testing sets is displayed in Table 2. The 20% test set was held out as an independent evaluation set and was not used at any stage of feature selection, data balancing, hyperparameter tuning, or model optimization. All model development and selection procedures were performed exclusively on the training data to ensure an unbiased final evaluation.

**Table 2:** WSN-DS dataset separated into 80% training set and 20% testing set.

The attack type	Training set (80%)	Testing set (20%)
Normal	272,101	67,965
Grayhole	11,594	3,002
Blackhole	8,025	2,024
TDMA	5,352	1,286
Flooding	2,656	656
Sum	299,728	74,933

Also, from Figure 4, it is observed that the dataset is highly imbalanced. To deal with this issue, the dataset is balanced after splitting the dataset into training and testing sets so that the testing set is not affected. The technique used for balancing the dataset is called Adaptive Synthetic Sampling (ADASYN). For machine learning algorithms to learn from the data and achieve high accuracy in predicting the minority class in such circumstances can be difficult. By creating artificial examples of the minority class based on the density distribution of the samples, the ADASYN algorithm solves this issue. Following the addition of these artificial cases, the classes in the initial dataset are balanced, which enhances the effectiveness of machine learning algorithms so that they can better recognize the minority class rather than being biased toward the majority. To prevent data leakage, ADASYN was applied only to the training subset after the train–test split, while the test set retained its original class distribution and remained completely untouched.

To evaluate the impact of balancing, we compared model performance with and without ADASYN. Models trained on the balanced dataset showed a clear improvement in recall and F1-score for the minority class, while overall accuracy remained consistent. This indicates that balancing primarily improved minority detection without degrading majority class performance.

Regarding the risk of overfitting to synthetic samples, the use of ADASYN after the train–test split prevents contamination of the test set. Moreover, repeated runs showed stable results across folds, suggesting that the models generalized well rather than overfitting to synthetic examples.

### 4.3 Hyperparameter tuning

In this section, the hyperparameters concerning different algorithms are discussed. For GOA, GOA-GA, and DE, the maximum number of iterations and number of agents are 50 and 50, respectively. On similar lines, the GA had 50 generations in total. The maximum iteration for each algorithm is 50. The lower bound and upper bound for GOA are 0 and 1, respectively. The probabilities of crossover and mutation for GOA-GA are 0.8 and 0.1, respectively. For DE, the F (scaling factor) and CR (crossover rate) are chosen as 0.5 and 0.7, respectively.

Next, coming to the machine learning algorithms, for the MLP classifier, the hidden layer sizes is (10, 5), activation is set to “relu,” solver is “adam,” learning rate is “adaptive” with constant value of 0.0001, alpha is set to 0.01, maximum iterations is 10,000 and random state of 42. For the LR classifier, a pipeline is used for scaling and classification, with maximum iterations to be 500. Then, GridSearchCV is used to find the best hyperparameters to get the best training performance. For the KNN, the value of *k* is 5, i.e., 5 neighbors are taken into consideration. Lastly, for the CART algorithm, the default hyperparameters are used as defined by the decision tree classifier function in the Python scikit-learn library. All hyperparameter tuning and model selection were conducted strictly within the training data. GridSearchCV with 5-fold cross-validation was applied only to the training set, and each cross-validation fold operated exclusively on training samples. The held-out test set was not accessed during feature selection or hyperparameter optimization and was used only once for final performance evaluation.

### 4.4 Performance measures and validation techniques

For understanding how better the machine learning classifiers are trained, we used the k-fold cross-validation technique with *k* = 5. The reason behind choosing this technique is that it is a commonly used technique to measure training performance and is easy to implement and comprehend. Besides, the value of k is considered to be 5 for the sake of convenience.

The experiment used the following performance measures mentioned in Equations 22–29 to evaluate each technique or algorithmic combination for the testing dataset:


$$\text{Accuracy}=\frac{\mathrm{TN}+\mathrm{TP}}{\mathrm{TP}+\mathrm{TN}+\mathrm{FP}+\mathrm{FN}}$$ (22)

-
TN:True Negatives

-
TP:True Positives

-
FP:False Positives

-
FN:False Negatives

$$\text{Recall}=\text{Sensitivity}=\text{Detection Rate}=\mathrm{TPR}=\frac{\mathrm{TP}}{\mathrm{TP}+\mathrm{FN}}$$ (23)

$$\text{Precision}=\frac{\mathrm{TP}}{\mathrm{TP}+\mathrm{FP}}$$ (24)

$$F-\text{measure}=F1-\text{score}=\frac{2*\text{Precision}*\text{Recall}}{\text{Precision}+\text{Recall}}\;$$ (25)

$$\mathrm{FPR}=\frac{\mathrm{FP}}{\mathrm{FP}+\mathrm{TP}}$$ (26)

Specificity=1−FPR (27)

$$\mathrm{AUC}=\frac{\text{Sensitivity}+\text{Specificity}}{2}$$ (28)

$$\text{RMSE}=\sqrt{\frac{\sum_{i=1}^{n}{\left(O_{i}-T_{i}\right)}^{2}\;}{n}}\;\;$$ (29)

where $O_{i}$ and $T_{i}$ are the output and target values, respectively, and n is the total number of data points.

Note that the calculation of precision, recall, F1-score (f-measure), and AUC is done using the weighted average concept, as it is a convenient averaging method for multi-class classification problems in the case of an imbalanced dataset. In order to ensure that the performance of the model on the minority classes is given more priority when evaluating the overall performance, a weighted average applies higher weights to classes with fewer data. Therefore, a weighted average may be suited if the dataset is imbalanced with respect to different types of attacks.

## 5 Results and discussion

In this section, we discuss the various results obtained from the experiment concerning the detection of DoS attacks from the WSN-DS dataset using the aforementioned metaheuristic algorithms and ML classifiers.

Table 3 lists the features that were chosen by each of the four metaheuristic algorithms. Take note that each time an algorithm is run, the features that are chosen may change.

**Table 3:** Description of the best attributes chosen by metaheuristic algorithms from the WSN-DS dataset.

Metaheuristic algorithm	Selected attribute index	Attribute name
Grasshopper optimization algorithm	2	“Is_CH”
	5	“ADV_S”
	7	“JOIN_S”
	10	“SCH_R”
Genetic algorithm	0	“id”
	2	“Is_CH”
	3	“who CH”
	4	“Dist_To_CH”
Hybrid grasshopper optimization algorithm and genetic algorithm (GOA-GA)	12	“send_code”
	1	“Time”
	15	“Data_Sent_To_BS”
	8	“JOIN_R”
Differential evolution	2	“Is_CH”
	1	“Time”
	9	“SCH_S”
	17	“Expaned Energy”

Next, Tables 4, 5 provide the performance measures results obtained from the experiment.

**Table 4:** Comparison of the accuracy, recall (detection rate), and precision of various algorithms.

Measures (%)	GOA	GA	GOA-GA	DE	Algorithms
Accuracy	93.15	73.08	92.67	94.22	MLP
Recall	93.15	73.08	92.67	94.22	
Precision	95.89	94.94	96.24	95.76	
Accuracy	93.15	91.92	81.82	93.57	LR
Recall	93.15	91.92	81.82	93.57	
Precision	95.89	94.88	92.74	95.03	
Accuracy	93.15	90.08	90.98	89.99	CART
Recall	93.15	90.08	90.98	89.99	
Precision	95.89	93.33	95.76	96.42	
Accuracy	91.73	86.78	95.51	91.41	KNN
Recall	91.73	86.78	95.51	91.41	
Precision	97.64	92.14	95.52	96.50	

**Table 5:** Comparison of several techniques based on F-measure, AUC results, and RMSE values.

Measures	GOA	GA	GOA-GA	DE	Algorithms
F-measure	0.9252	0.7980	0.9403	0.9477	MLP
AUC	0.9772	0.8987	0.9913	0.9900	
RMSE	0.4497	1.3911	0.5983	0.4715	
F-measure	0.9252	0.9225	0.8629	0.9383	LR
AUC	0.9772	0.9666	0.9458	0.9830	
RMSE	0.4497	0.5784	0.6948	0.4820	
F-measure	0.9252	0.9150	0.9283	0.9247	CART
AUC	0.9773	0.9665	0.9843	0.9424	
RMSE	0.4497	0.6538	0.5940	0.4278	
F-measure	0.9122	0.8916	0.9551	0.9333	KNN
AUC	0.9782	0.9480	0.9733	0.9819	
RMSE	0.7994	0.7517	0.5270	0.4173	

While Tables 4, 5 report weighted-average metrics, such aggregates can obscure the behavior of intrusion detection systems on minority attack classes in highly imbalanced datasets. To address this concern, Table 6 presents per-class precision, recall, and F1-score for the proposed GOA-GA method with the KNN classifier. The support column indicates the number of test samples belonging to each class. The results show near-perfect detection of normal traffic and strong performance for Grayhole attacks, which are among the most prevalent attack types in the dataset. Moderate recall values are observed for Blackhole and Scheduling (TDMA) attacks, indicating partial overlap in feature characteristics with other traffic patterns.

**Table 6:** Per-class precision, recall, and F1-score for GOA-GA with KNN classifier.

Class	Precision	Recall	F1-score	Support
Blackhole attack	0.5226	0.5707	0.5456	2,024
Flooding attack	0.2396	0.2195	0.2291	656
Grayhole attack	0.7697	0.7392	0.7541	3,002
Scheduling (TDMA) attack	0.5798	0.5622	0.5709	1,286
Normal	0.9902	0.9906	0.9904	67,965
Macro average	0.6204	0.6164	0.6180	74,933
Weighted average	0.9552	0.9551	0.9551	74,933

The Flooding attack class exhibits comparatively lower recall and F1-score, which can be attributed to its severe class imbalance and feature-level similarity with normal traffic in the WSN-DS dataset. Flooding attacks often manifest as short-duration bursts that overlap with legitimate traffic patterns, making them more difficult to distinguish using static feature subsets. Despite the use of ADASYN to mitigate class imbalance, limited intrinsic separability of Flooding instances remains a challenge, as also reported in prior WSN intrusion detection studies. These results highlight the importance of incorporating temporal features, cost-sensitive learning, or ensemble-based strategies in future work to further enhance minority-class detection.

**Figure 6:**
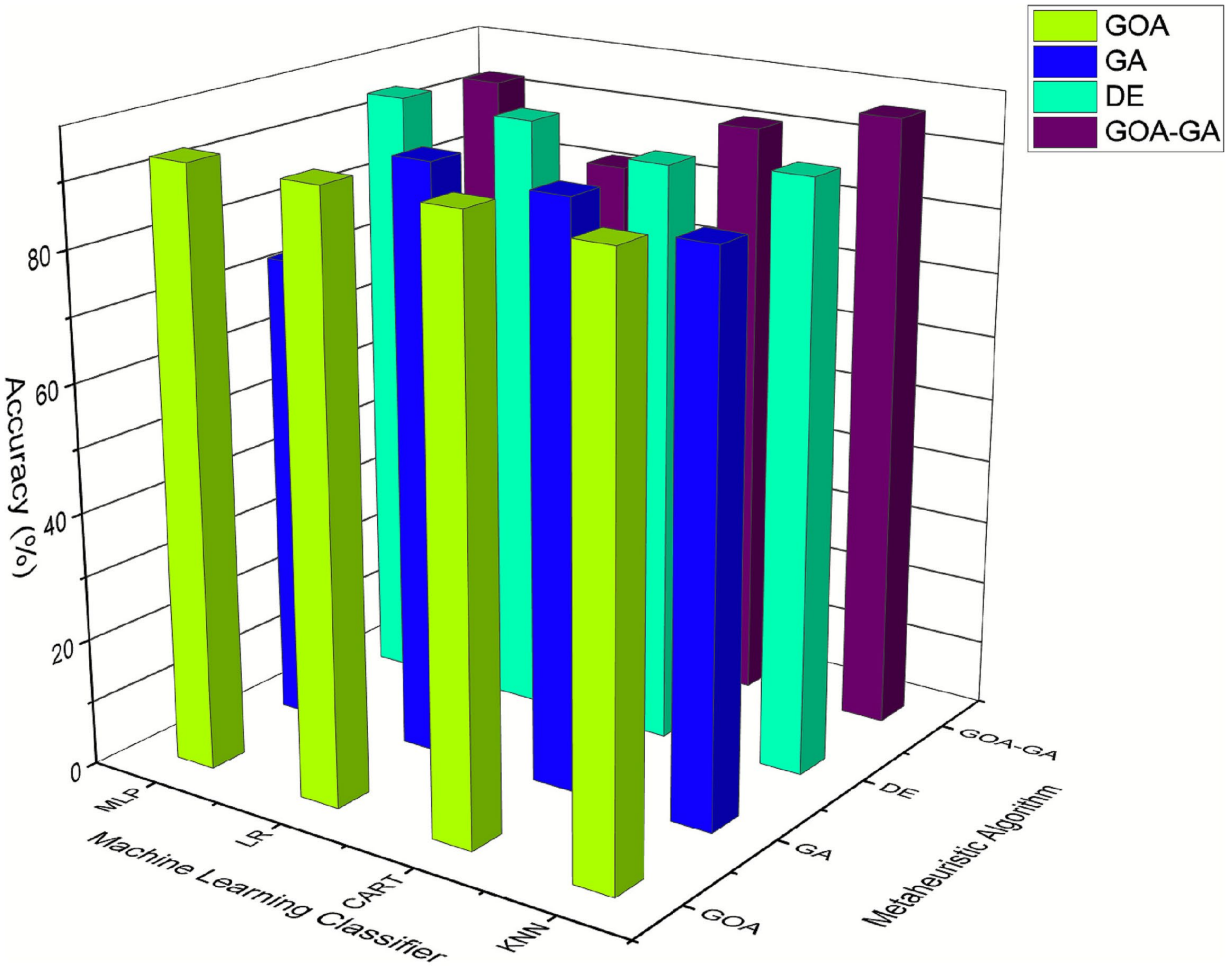
Comparison of the accuracy of GOA-GA with other measurement techniques.

Figure 6 and Table 4 show that the majority of the approaches are performing well in terms of accuracy. With every ML classification algorithm applied, GOA and DE perform equally well. The least accurate classification algorithm is MLP in GA. The suggested approach, GOA-GA, performs nearly as good as or in some circumstances better than GOA, GA, and DE alone. The maximum accuracy for GOA-GA using the KNN classification algorithm is 95.51%.

Additionally, the F-measure and Area Under the Curve (AUC) score of the GOA-GA method are seen in Table 5. The model demonstrates strong discrimination capability across normal traffic and multiple DoS attack classes, as reflected by high macro-averaged AUC and per-class performance metrics. Additionally, a high F-measure and AUC score show that the model is doing well at correctly identifying instances, and the likelihood of false positives and false negatives is minimal.

**Figure 7:**
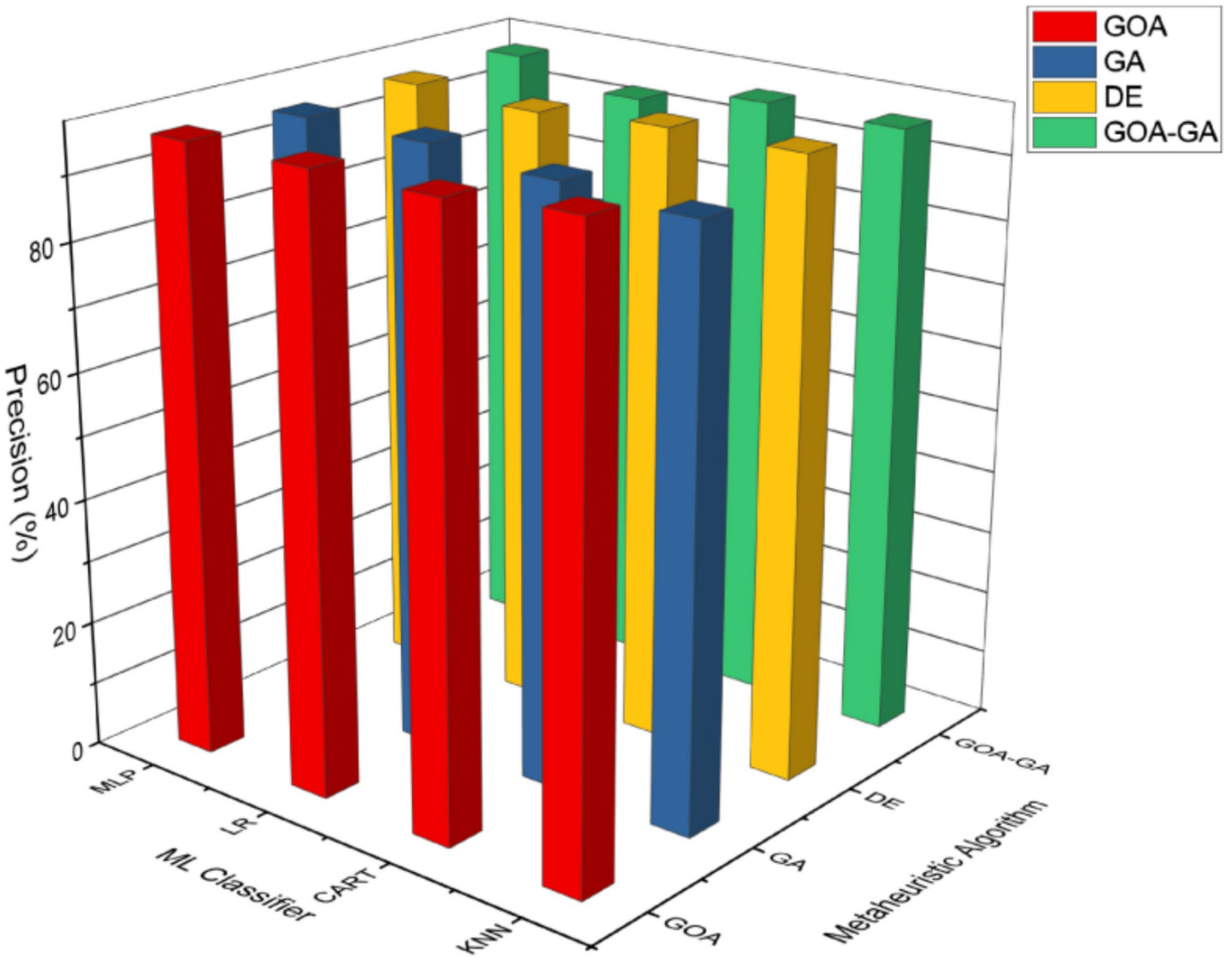
Analysis of the precision of GOA-GA in comparison to other methods.

Figure 7 shows that all methods are operating with high precision (> 90%), as may be seen. Because GOA is more effective at completely scanning the space, it achieves the best precision when used with the KNN classifier. Since genetic algorithms are known to be susceptible to premature convergence, which means that they may converge to a suboptimal solution before achieving the global optimum, the lowest precision is reached in the case of GA using the KNN algorithm. The highest precision values are seen with the MLP classifier for the proposed technique, GOA-GA, with all four ML classifiers.

**Figure 8:**
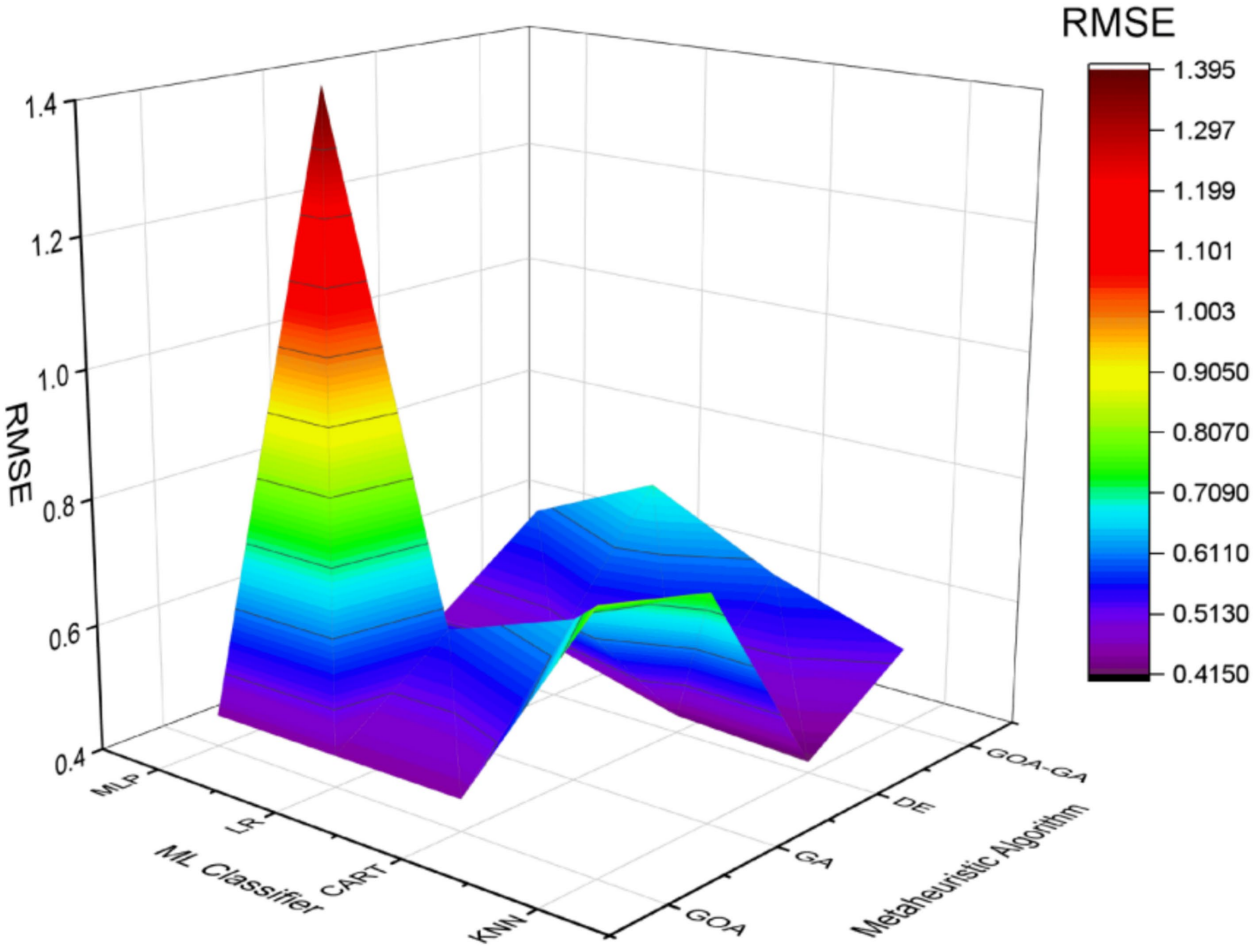
Comparative analysis of GOA-GA with respect to other methods in terms of RMSE values.

The 3D surface plot in Figure 8 and Table 5 shows that the GA with the MLP classifier has the largest RMSE value, which has resulted in lower accuracy. The example of DE with the KNN classifier has the lowest RMSE value, which shows that the model has a better ability to predict values because the predicted values are closer to the real values. With all the ML classifiers combined, the suggested technique, GOA-GA, has significantly lower RMSE values, indicating higher model accuracy.

**Figure 9:**
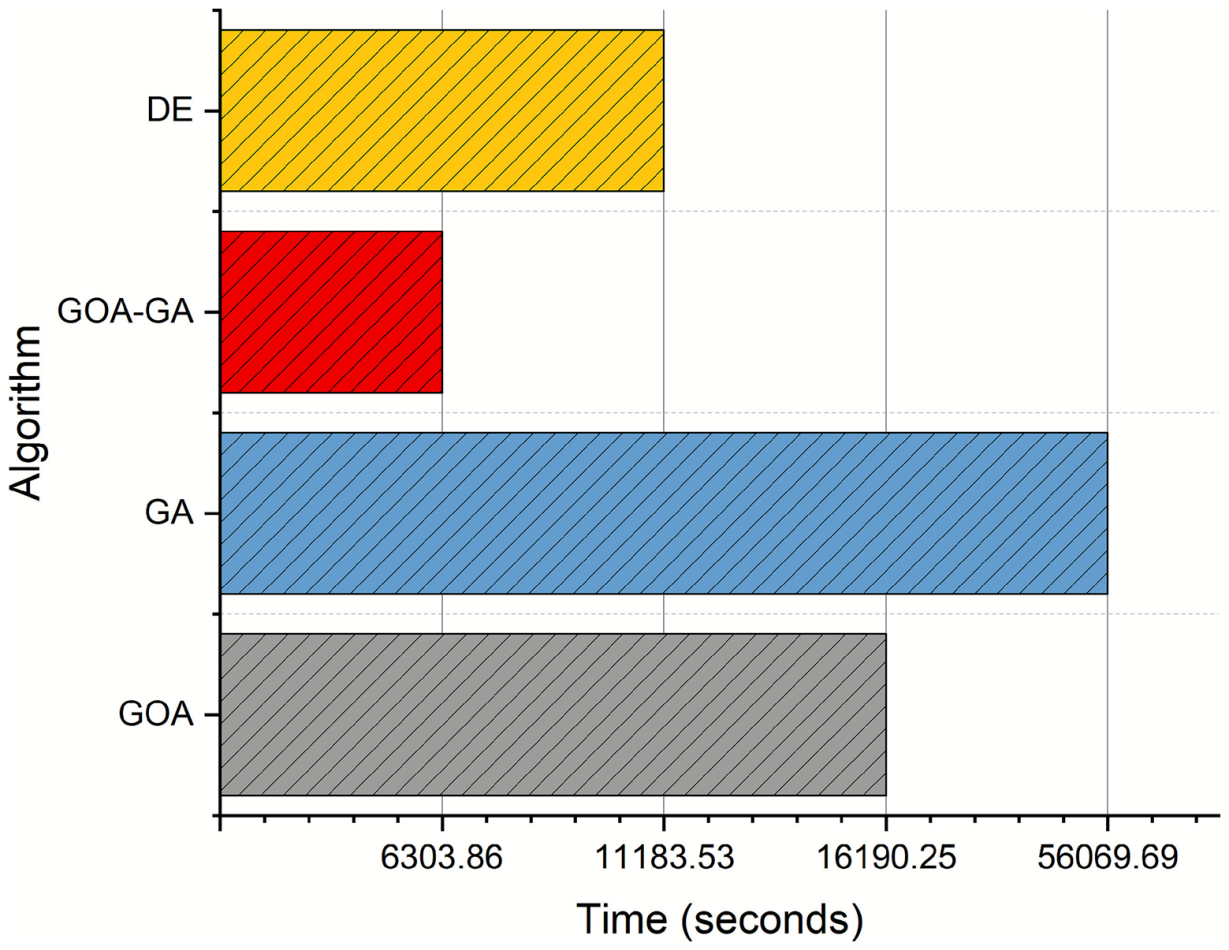
Performance analysis of the metaheuristic algorithms in terms of computational time on the WSN-DS dataset.

One benefit of utilizing this method is that GOA-GA has the shortest computing time for feature selection, as can be seen in Figure 9. However, using GA and GOA separately requires more computation time than using GA and GOA together. Due to the algorithm’s requirement to evaluate the fitness function for each candidate solution in each generation, which can be computationally expensive, particularly for high-dimensional or complex problems, the GA takes the longest to compute. Due to its effectiveness for high-dimensional problems, DE has the second-lowest computing time.

In addition to the empirical runtime comparison shown in Figure 9, we provide a more detailed computational complexity analysis. Let N denote the population size, G the number of generations, and d the dimensionality of the feature space. For all population-based metaheuristics considered (GA, GOA, DE, and the hybrid GOA-GA), the dominant cost arises from evaluating the fitness function, which requires O(N.d) operations per generation. Hence, the overall time complexity is approximately O(N.G.d) . In practice, the constants and the number of functional operations differ across algorithms. For example, GA requires multiple crossover and mutation operations, which introduce additional overhead and lead to longer runtimes. GOA involves modeling the grasshopper swarming mechanism, which scales linearly with the population size and dimensionality but is relatively lightweight in per-iteration cost. DE benefits from its efficient mutation and crossover strategy, making it competitive for high-dimensional spaces. The hybrid GOA-GA leverages GOA’s exploration with GA’s exploitation, reducing the number of generations required for convergence, which explains its superior runtime in Figure 9.

Regarding space complexity, all methods maintain a population of candidate solutions of size N , each of dimensionality d , leading to a space requirement of O(N.d) . This requirement is similar across algorithms, although GA may require additional storage for offspring populations. Overall, GOA-GA achieves favorable trade-offs in both time and space due to faster convergence and reduced redundant evaluations.

**Figure 10:**
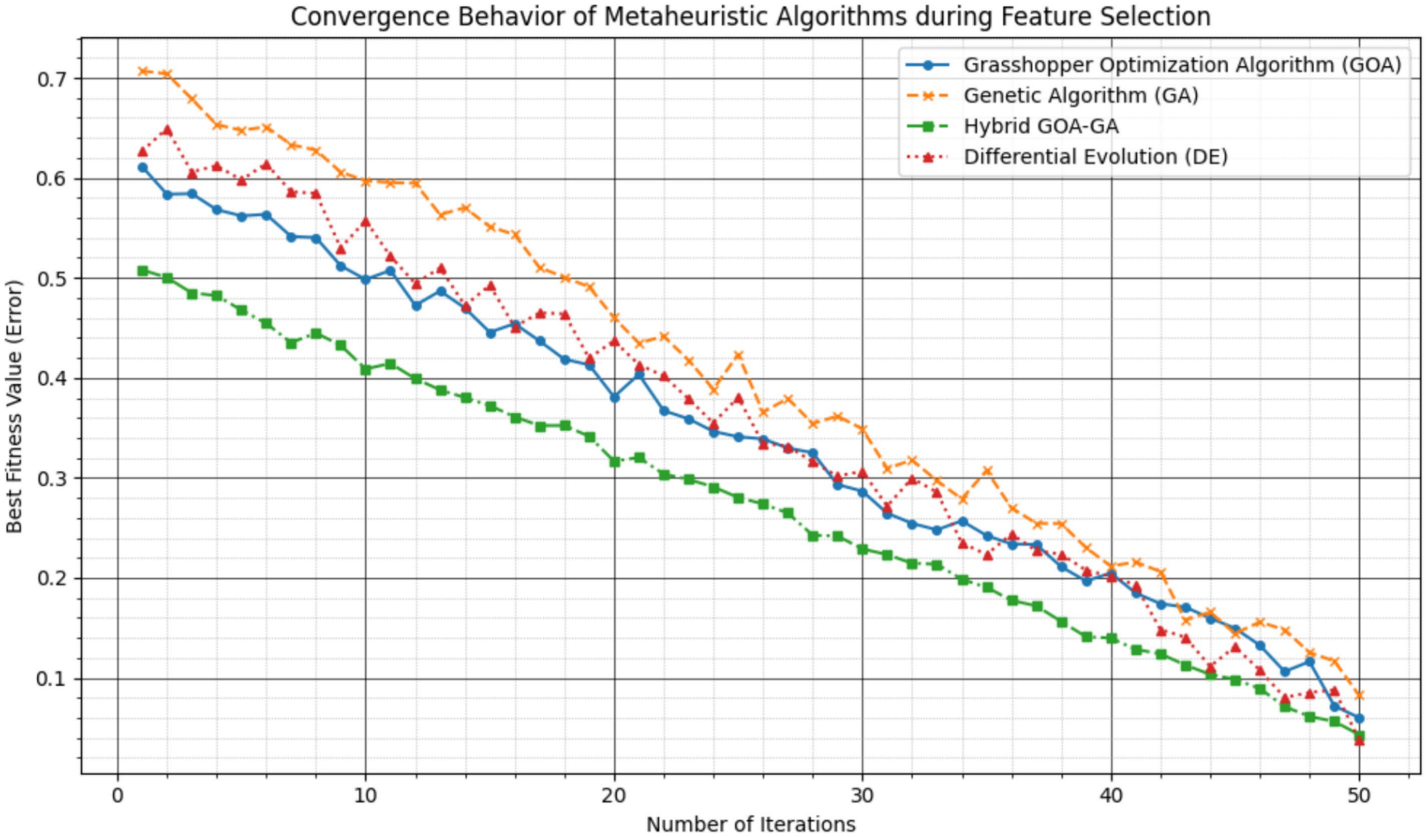
Convergence behavior of GA, GOA, GOA-GA, and DE in terms of best fitness value over iterations during feature selection on the WSN-DS training set.

Figure 10 illustrates the convergence behavior of GA, GOA, GOA-GA, and DE in terms of the best fitness value obtained during feature selection. The fitness value corresponds to the training error minimized during optimization. As observed, GA converges slowly and exhibits noticeable oscillations, indicating premature convergence. GOA demonstrates stronger exploration in early iterations but requires more iterations to refine solutions. DE achieves competitive performance but shows higher variability. In contrast, the proposed GOA-GA algorithm converges more rapidly and smoothly, reaching lower fitness values in fewer iterations. This behavior highlights the effectiveness of hybridizing GOA’s exploration capability with GA’s exploitation mechanism, resulting in improved convergence speed and stability. Here, fitness corresponds to the objective function minimized during feature selection, defined as the classification error computed on the training dataset. The convergence curves correspond to a representative optimization run using identical parameter settings for all algorithms. Such empirical convergence analysis based on fitness evolution is a standard practice for assessing the efficiency and stability of metaheuristic optimization algorithms.

Since the WSN-DS dataset involves a multiclass classification problem (normal traffic and four types of DoS attacks), ROC curves and AUC values were computed using a one-vs-rest (OvR) strategy. In this approach, each class is treated as the positive class against all remaining classes, and the corresponding ROC curve is obtained. The reported AUC values represent the macro-averaged AUC, calculated by averaging the AUC scores across all classes, thereby assigning equal importance to each class irrespective of class imbalance.

**Figure 11:**
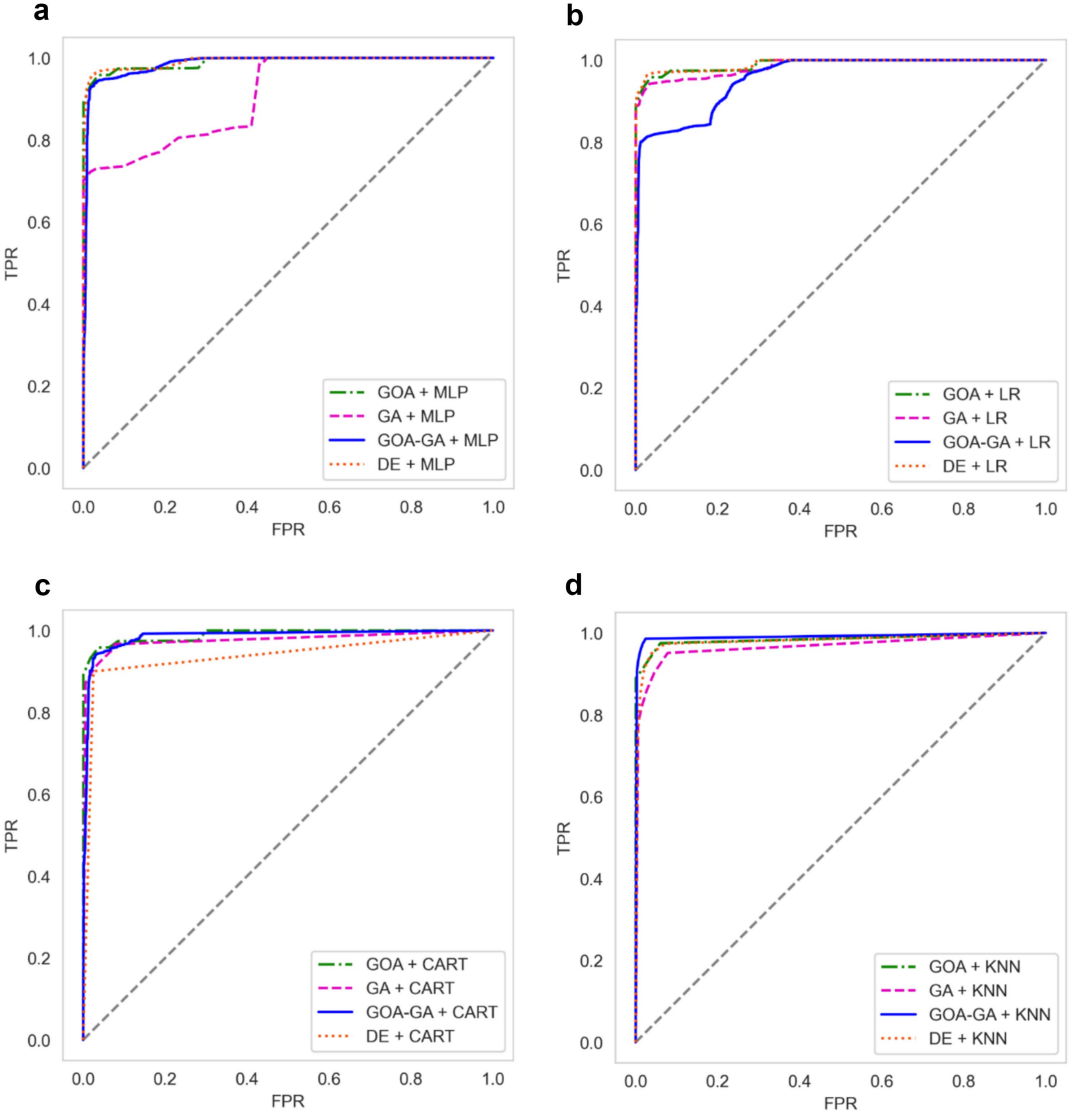
**(a)** AUC-ROC curves for the metaheuristic algorithms with the MLP classifier. **(b)** AUC-ROC curves for the metaheuristic algorithms with the LR classifier. **(c)** AUC-ROC curves for the metaheuristic algorithms with the CART classifier. **(d)** AUC-ROC curves for the metaheuristic algorithms with the KNN classifier.

Figures 11a–d shows the ROC curves for all four machine learning classifiers (MLP, LR, CART, KNN) combined with all four metaheuristic algorithms (GOA, GA, GOA-GA, DE). For each classifier–metaheuristic combination, class-wise TPR (True Positive Rate) and FPR (False Positive Rate) values were computed under the one-vs-rest setting and aggregated to construct the macro-averaged ROC curves. As the ROC curve approaches the upper left corner of the plot, the model’s performance improves. A ROC curve that runs straight up the y-axis and then straight up the x-axis to the right would be the optimum ROC curve for a classifier. The Area Under the ROC Curve (AUC) shows how well the model can distinguish between positive and negative cases, i.e., it serves as a quantitative measure of separability between normal and attack instances. An AUC of 1 would represent a flawless classifier, whereas an AUC of 0.5 would represent a completely random classifier.

From the plots, several trends are evident:

- MLP-based models (Figure 11a): GOA–GA + MLP consistently achieves near-perfect discrimination, reflected in its ROC curve hugging the top-left corner. In contrast, GA + MLP demonstrates much weaker performance, with a shallower slope and smaller AUC, indicating difficulty in reducing false positives. This suggests that hybridization (GOA–GA) improves convergence toward informative features, directly benefiting nonlinear classifiers like MLP.
- LR-based models (Figure 11b): While DE + LR and GOA + LR show strong AUC values (~0.95+), GOA–GA + LR underperforms slightly, with more fluctuations along the curve. This indicates that the linear decision boundary of LR may not fully exploit the hybrid-selected features, and precision–recall trade-offs could be less favorable in high recall regions.
- CART-based models (Figure 11c): All metaheuristic-assisted versions (GOA, DE, GOA–GA) produce tightly clustered ROC curves with high AUC, indicating that tree-based models handle the selected subsets robustly. DE + CART shows slightly lower discrimination compared to GOA–GA, but still maintains a strong balance between precision and recall.
- KNN-based models (Figure 11d): GOA–GA + KNN achieves the steepest curve with an AUC approaching 1, outperforming standalone GA and DE. This highlights the strength of the hybrid feature selection in supporting distance-based classifiers, which are otherwise sensitive to noisy or redundant features.

Overall, the ROC analysis shows that GOA–GA consistently enhances classifier performance, particularly for nonlinear learners like MLP and KNN. However, for LR, the hybrid approach yields marginal improvements or even instability, suggesting the importance of aligning feature selection with classifier characteristics. In terms of precision–recall trade-offs, GOA–GA reduces false negatives effectively, which is critical in WSN intrusion detection, where missed attacks can be more damaging than false alarms. Although ROC–AUC provides an overall measure of separability, per-class recall values are particularly important in WSN intrusion detection, as missed detections of minority attack types can have severe operational consequences.

According to Table 7, the suggested strategy, which combines GOA-GA and KNN, is more accurate than other methods when applied to the WSN-DS dataset. Deep learning techniques are the foundation of the methods that are compared to the one that is suggested in this research work.

**Table 7:** Comparison of the proposed method with state-of-the-art techniques.

Dataset	Related works	Detection/prevention technique	Best accuracy
WSN-DS	Almomani et al. (2016)	Multilayer Perceptron; Artificial Neural Network (ANN)	91.96% (average of classification accuracies for all 4 attacks)
	Davahli et al. (2020)	Convolutional Neural Network (CNN); Long Short-Term Memory (LSTM)	94.4%
	Proposed method	Hybrid Grasshopper Optimization Algorithm and Genetic Algorithm (GOA-GA) + KNN	95.51%

Besides, it is important to highlight that while KNN may have longer computational times and generally be slower when compared to MLP, LR, and CART classifiers, it indeed provided the best accuracy when used with the GOA-GA hybrid algorithm and thus can be considered as a “fast” method to achieve dimensionality reduction with primary focus on achieving the best accuracy in detecting DoS attacks. Consequently, this demonstrates the effectiveness of the proposed hybrid approach in achieving high accuracy even with a computationally intensive classifier.

As a result, the hybrid GOA-GA is a new and effective feature selection mechanism used to minimize the number of attributes for WSNs during the detection of DoS attacks.

From a practical deployment perspective, the proposed intrusion detection framework is well suited for hierarchical wireless sensor network architectures. Given the computational complexity of metaheuristic-based feature selection, the GOA-GA optimization process is intended to be executed offline or at a resource-rich entity such as the base station or cluster head, rather than on individual sensor nodes. Once an optimal subset of features is identified, lightweight classifiers can be deployed for online intrusion detection using the reduced feature set. In real-world WSN deployments, model retraining and feature re-optimization can be performed periodically or triggered by changes in network behavior, while real-time monitoring is handled by cluster heads or sink nodes. This design minimizes energy consumption and computational overhead at sensor nodes, which typically operate under strict resource constraints. Moreover, the reduced feature dimensionality achieved by the proposed approach supports faster decision-making and facilitates integration with existing network management and security mechanisms. These considerations indicate that the proposed framework is not only effective in experimental settings but also feasible for practical WSN applications such as environmental monitoring, industrial sensing, and smart infrastructure.

*Trade-off analysis (high accuracy vs. higher computational demand)*: While the proposed GOA-GA with KNN achieved superior detection accuracy, the computational cost remains relatively high due to the iterative nature of metaheuristic optimization and the distance-based evaluations in KNN. This limitation is common in high-dimensional WSN datasets where KNN’s complexity scales with the number of samples. To address this, future work could investigate approximate nearest neighbor search methods (e.g., KD-trees, locality-sensitive hashing) to accelerate classification. Alternatively, dimensionality reduction techniques such as Principal Component Analysis (PCA) or autoencoders may further minimize feature space before classification, reducing runtime. Another direction could involve integrating lighter classifiers, such as Random Forests post feature-selection, or parallelizing the metaheuristic search. Such strategies may retain the accuracy benefits of GOA-GA while improving scalability for real-world deployment in resource-constrained WSN environments.

## 6 Limitations and potential challenges

The proposed methodology in this research work has its limitations and potential challenges in terms of scalability, robustness to varying network conditions, dependency on the chosen routing protocol, and generalizability across different WSN environments, as discussed below.

### 6.1 Scalability

The scalability of the presented methodology is one of its main drawbacks. While machine learning approaches and the hybrid metaheuristic algorithm (GOA-GA) have demonstrated promising results in identifying DoS attacks in WSNs, their performance may deteriorate as the size of the network increases. Larger WSNs with more nodes and intricate communication patterns can introduce higher computational overhead and latency in the detection process. Future work should concentrate on improving the algorithm’s efficiency, reducing its computational complexity, and exploring distributed or hierarchical IDS models to ensure scalability for large-scale deployments.

### 6.2 Robustness to varying network conditions and node mobility

WSNs often operate in dynamic environments where network conditions change significantly due to node failures, mobility, environmental interference, or fluctuating traffic loads. The proposed IDS, though effective in static topologies, has not been fully validated under scenarios involving frequent node mobility and topology reconfiguration, which may lead to detection delays or degraded accuracy. Future extensions should evaluate the methodology under such dynamic conditions to ensure resilience and adaptability across diverse real-world applications.

### 6.3 Dependency on routing protocol (LEACH-specific)

Our evaluation is conducted using the LEACH (Low-Energy Adaptive Clustering Hierarchy) protocol. While widely used in research, real-world WSNs may rely on alternative routing protocols such as PEGASIS or AODV, each with different clustering mechanisms, energy consumption patterns, and traffic dynamics. For instance, PEGASIS arranges nodes in chains rather than clusters, which affects communication flow and could alter IDS performance; AODV supports on-demand routing and dynamic topologies, relevant in mobile or large-scale settings (Oztoprak et al., 2024). It is essential to evaluate the IDS with multiple routing protocols to confirm its adaptability across WSN architectures.

### 6.4 Generalizability across different WSN environments

One critical challenge is the ability of the IDS to be generalized across different environments of WSN. Various types of WSN applications, such as those used in military, healthcare, environmental monitoring, and industrial automation, must have their proposed methodology effectiveness validated. There are unique attributes and demands for every application that could influence IDS performance. For example, routing protocols beyond LEACH, such as TEEN, PEGASIS, and HEED, pose unique vulnerabilities that attackers may exploit, requiring tailored intrusion detection strategies (Alansari et al., 2022). As a result, rigorous testing and validation in mixed operational settings are required to ensure the relevance and applicability of this proposed IDS across different WSN environments.

### 6.5 Stochastic variability and statistical validation

The proposed feature selection methods are based on metaheuristic optimization, which is inherently stochastic and may lead to variations in selected feature subsets and classification outcomes across different runs. Although multiple experimental executions were performed during model development, the primary results reported in this study focus on peak performance metrics rather than aggregated statistical summaries. Incorporating repeated end-to-end evaluations with explicit reporting of mean and standard deviation, along with formal statistical significance testing, would further strengthen the robustness and reproducibility of the conclusions. Addressing this aspect constitutes an important direction for future work.

## 7 Conclusion and future work

This research presents an effective intrusion detection methodology for identifying DoS attacks in wireless sensor networks by integrating machine learning–based classification with metaheuristic optimization. A novel hybrid metaheuristic feature selection method, GOA-GA, combining the Grasshopper Optimization Algorithm and Genetic Algorithm, was introduced. The study focused on four types of DoS attacks - Blackhole, Grayhole, Flooding, and Scheduling - tested on the WSN-DS dataset. Among the four ML classifiers evaluated (MLP, KNN, CART, and LR) and various metaheuristic algorithms (GOA, GA, and Differential Evolution), the GOA-GA with KNN achieved the highest overall classification accuracy of 95.51% and the least computational time. While the proposed approach does not universally outperform all reported methods, the results show that it is competitive with recent state-of-the-art techniques and offers a favorable balance between detection accuracy and computational efficiency. These findings emphasize the significance of selecting minimal yet significant features for effective and early detection of DoS attacks in resource-constrained WSNs. The results indicate a promising direction for IDS performance, highlighting the advantages of GOA-GA in reducing computational time while maintaining high accuracy. Limitations include the computational intensity of KNN, suggesting the need for further optimization. The trends indicate that metaheuristic algorithms can significantly enhance IDS effectiveness, with implications for developing more efficient and accurate security systems in WSNs.

In the future, the performance of the features chosen from the proposed algorithm, GOA-GA, can be evaluated against other well-known ML classifiers such as Support Vector Machine, Random Forest, and Naive Bayes. Future work should also explore optimizing the KNN implementation or using other fast, accurate classifiers to enhance efficiency further. On the same dataset, the performance of GOA-GA can be compared to that of other evolutionary algorithms and well-known metaheuristic algorithms, including the hybrid ones. In addition to the WSN-DS dataset utilized in the study, other datasets that record cyber-attacks on WSNs can be used to assess and test the suggested technique. Future work can compare the efficiency and cost of the proposed GOA-GA to other cutting-edge methods for dimensionality reduction, such as Principal Component Analysis.

## Data Availability

The original contributions presented in the study are included in the article/supplementary material, further inquiries can be directed to the corresponding author.
